# Understanding barriers and enablers for vaccination against COVID-19 and influenza among healthcare workers: a mixed-methods study nested within the UK SIREN cohort

**DOI:** 10.1136/bmjopen-2025-113889

**Published:** 2025-12-17

**Authors:** Dominic Sparkes, Katie Munro, Atiya Kamal, Jack Haywood, Anna Howells, Sarah Foulkes, Sophie Russell, Naomi Platt, Jonathan Broad, Colin S Brown, Susan Hopkins, Jasmin Islam, Victoria Hall, SIREN Study Team

**Affiliations:** 1 UKHSA, London, UK; 2 School of Social Sciences, Birmingham City University, Birmingham, UK; 3 Public Health Wales, Cardiff, UK; 4 SIREN Study Team, UK Health Security Agency, London, UK

**Keywords:** COVID-19, Vaccination, VIROLOGY

## Abstract

**Objectives:**

To investigate vaccination coverage for influenza and COVID-19 in the SARS-CoV-2 immunity and reinfection evaluation (SIREN) study cohort of healthcare workers (HCWs) between 2020 and 2023 and explore vaccination enablers and barriers.

**Design:**

A mixed-methods study nested within SIREN, a multicentre prospective cohort study of HCWs across the UK. Quantitative and qualitative methods were used sequentially, using an expansion/explanation function, enabling emergent themes observed from the quantitative stage to be explored in the qualitative stage.

**Setting:**

SIREN sites include secondary care centres and community mental health trusts in the UK.

**Participants:**

Quantitative analysis was conducted on data from 6048 participants. Participants were representative of the HCW workforce, with the majority being women (83%) and of white ethnicity (88%). Nurses made up the largest occupational group (33%). Qualitative analysis of data from 24 participants including five focus groups (n=21) and three semistructured interviews (n=3); 82% women, 26% minority ethnic, all working age from across the UK.

**Primary outcome measures:**

Quantitative: vaccine coverage for COVID-19 and influenza vaccines by demographic with multivariable logistical regression used to assess differences. Qualitative: thematic analysis to explore reasons behind the results seen in the quantitative stage.

**Results:**

COVID-19 vaccination was initially high; 97% received two doses and 94% a first booster. However, coverage was reduced to 77%, for the second booster. Influenza vaccination coverage was lowest in 2020–2021 (46%), increasing to 73% in 2021–2022 and to 79% in 2022–2023. In 2022–2023, vaccination coverage was higher for influenza than for COVID-19. High vaccine coverage for both COVID-19 and influenza was observed in doctors, pharmacists and therapists. Porters, healthcare assistants and staff from minority ethnic groups had lower vaccine coverage for both COVID-19 and influenza. Four themes were identified: (1) attitudes towards vaccination changed throughout the COVID-19 pandemic; (2) HCWs used data to inform vaccination decisions; (3) poor communication in healthcare settings contributed to a reduction in vaccination; (4) there were both positive and negative impacts of the COVID-19 vaccine on influenza vaccine uptake and other vaccination programmes.

**Conclusions:**

Between 2020 and 2023 in our cohort, COVID-19 vaccination coverage decreased, whereas influenza increased. Our study found attitudes to both vaccines have shifted, becoming more favourable to influenza and less to COVID-19 boosters. Barriers to COVID-19 boosters, including concerns about side effects and vaccine effectiveness, need to be addressed with improved communication on the benefits and adverse events. Future vaccination strategies should address the differences we have identified in vaccine coverage across demographics and occupational groups, including continued efforts to improve vaccine equity.

**Trial registration number:**

ISRCTN11041050.

**STRENGTHS AND LIMITATIONS OF THIS STUDY**

- This is a mixed-methods design nested within a prospective cohort study, which allowed us to rapidly explore trends we noticed in our cohort study data directly with our participants to help understand possible reasons for the differences in vaccine coverage observed.
- The scale of this study, nested within the SARS-CoV-2 immunity and reinfection evaluation (SIREN) study, allowed the inclusion of over 6000 participants followed for 3 years to see how vaccine coverage changed over the course of the COVID-19 pandemic.
- Being nested in the SIREN study cohort allowed additional information from a well-characterised cohort, including occupation, to be studied, which is not available in routinely collected vaccine coverage data and provides new insights.
- Key limitations from the vaccine coverage analysis include the representativeness of our cohort, with relatively small numbers in key occupational and minority ethnic groups, impacting the precision of our estimates in these groups and the generalisability of our findings to the wider healthcare workforce with a notably lower vaccination coverage.
- The participants are selected from the SIREN cohort and participants who engage in research may not be representative of the workforce as a whole and may also be influenced by their experiences as a research participant.

## Introduction

 The unprecedented development and deployment of COVID-19 vaccines is a powerful reminder that vaccination remains the most effective method of preventing infectious diseases.^1^,^3^ Barriers to vaccination can result in infection, illness and transmission. Understanding the factors that enable vaccine uptake and the barriers that prevent it is essential to informing vaccination programmes.^4 5^ Healthcare workers (HCWs) were prioritised in the COVID-19 vaccination programme, recognising both their occupational exposure and their role caring for vulnerable patients.^6^ Similarly, front-line HCWs are recommended to receive the annual influenza vaccine with significant efforts to increase coverage made over the last 20 years.^7^ HCWs hold a unique role within vaccination programmes as both important recipients and champions for their patients and communities, and in the prevention of nosocomial disease transmission.^8^ Their attitude towards vaccination is therefore vital for any successful vaccine roll-out. During the initial roll-out of COVID-19 vaccines in the UK, uptake among HCWs was unprecedented, with 91.2% of HCWs receiving at least one dose and 88.5% receiving two, but with declining uptake for third and subsequent doses by February 2022.^9^

During the COVID-19 pandemic, some population groups were disproportionately affected by COVID-19. Among HCWs, roles associated with direct access to patients (including porters and ancillary staff) were at an increased risk of contracting COVID-19.^4 10 11^ More widely, minority ethnic groups were also noted to be at an increased risk of infection and mortality.^412^,^14^ Given the critical role vaccination played in reducing both infections and mortality, it is important to understand any barriers to vaccine uptake within these groups to inform future deployment.^15^

The SARS-CoV-2 immunity and reinfection evaluation (SIREN) study is a prospective cohort study of HCWs in NHS hospitals across the UK, which investigates immunity to SARS-CoV-2 following infection and COVID-19 vaccination.^16^ The SIREN cohort, with an initial cohort of 44 543 HCWs includes doctors, nurses, healthcare assistants, therapists, midwives, pharmacists, porters and staff in administration and estates, allowing investigation into trends across the healthcare workforce.^16^

The aims of this work were to first compare trends in COVID-19 and influenza vaccine coverage throughout the pandemic in the SIREN cohort and then use this information to explore HCW perceptions of barriers and facilitators to vaccination, using a sequential mixed methods approach to give a unique insight into the views of healthcare professionals in the UK.

## Methods

### Study design

A mixed-methods study nested within the SIREN multicentre prospective cohort study of HCWs across the UK was used.^16^ Quantitative and qualitative methods were used sequentially, using an expansion/explanation function, enabling emergent themes observed from the quantitative stage to be explored in the qualitative stage through focus groups and thematic analysis.^17^ In line with improving standards of transparency in qualitative research, we have reported the qualitative section of the manuscript according to the Consolidated criteria for Reporting Qualitative research checklist (online supplemental table 1).^18^ Quantitative and qualitative data were connected, allowing emergent themes to be analysed sequentially.^19 20^

### Quantitative stage

The objective of the quantitative analysis was to describe COVID-19 and influenza vaccine coverage by time and demographic factors in the SIREN cohort, and compare this with national data on HCW vaccination coverage in the same period.

### Participants

Individuals were included in this analysis if they were enrolled in SIREN and had consented for active follow-up in March 2023. Participants were excluded if they had received their first COVID-19 vaccination before 07 December 2020 (ie, as part of a vaccine trial) or had withdrawn.

### Participant consent

The study protocol was approved by the Berkshire Research Ethics Committee on 22 May 2020, and the qualitative substudy was approved as Amendment 25 on 27 February 2023. Written consent was obtained from all participants on enrolment into the SIREN study. Focus group participants provided additional consent, as per the qualitative study amendment.

### Data collection

COVID-19 and influenza vaccine data (date and type) were obtained directly from participants via fortnightly follow-up surveys and through consented linkage on personal identifiers including name, date of birth and NHS number to the National Immunisation Management System (NIMS) (or equivalent system for the devolved administrations). The follow-up survey is included in online supplemental materials, only a small part of the questionnaire is used to confirm vaccination status rather than attitudes to vaccination. Self-reported demographic data, including age, ethnicity and job role, were obtained from SIREN enrolment questionnaires sent between June 2020 and April 2021.^16^ National surveillance data on HCW vaccination uptake in England were obtained from the annual UK Health Security Agency official statistics.^7^

### Data analysis

Vaccine coverage (the proportion of the cohort vaccinated) was calculated for each of the four COVID-19 vaccine doses, and for each influenza season (defined as 01 September until 31 March each year, in line with dates HCWs can receive each vaccine) between September 2020 and March 2023 (three seasons). For COVID-19 boosters, the first booster season was defined as 01 September 2021 to 31 March 2022, and the second booster as 01 September 2022 to 31 March 2023.

We described vaccination coverage over time, by dose/season and by demographic factors. We compared SIREN data with national data on HCWs in England. A multivariable logistic regression model, including age group, gender, ethnic group and occupation, was used to calculate adjusted ORs (aORs) and compare vaccine coverage across groups. STATA V.17 was used for data analysis and R V.4.3.1 was used for graphical presentation.

### Qualitative stage

The objective of the qualitative stage was to explore findings from the quantitative stage and identify potential barriers and enablers for COVID-19 and influenza vaccination among HCWs through a series of focus groups and interviews.

### Participants

Purposive sampling was used to recruit participants. Focus group and interview participants were all HCWs, including those in clinical and non-clinical roles (eg, administrators) and were recruited from three groups: SIREN participants, SIREN site research teams and wider workforce representatives at SIREN sites (including infection prevention and control teams and occupational health). Recruitment details and invitations to participate were advertised in the SIREN participant newsletter, the SIREN site team newsletter and the SIREN participant involvement panel (PIP), a panel of 10 participants, selected to represent cohort diversity (including region, job role, age, gender and ethnicity).^21^ Eligibility to join the focus groups and interviews was restricted to SIREN participants and site teams that were in active follow-up at the time of recruitment in March 2023, or a member of staff at any site where the SIREN study was taking place. Included participants were screened to ensure adequate representation from different occupations, regions, ethnicities and ages.

### Data collection

Qualitative data were collected via focus groups and semi-structured interviews, which were conducted online via MS Teams by two researchers with experience facilitating focus groups (AK and JH) independent of the co-ordinating SIREN Study Team in January–February 2023. Topic guides were developed by both the independent researchers and members of the co-ordinating SIREN Study Team. They were created to focus discussions and included several topics including testing, workplace absence and vaccination. Vaccination questions utilised preliminary findings on SIREN cohort vaccine coverage from years 2020–2022 from the quantitative stage. As part of the topic guide, participants were provided with some of this vaccine coverage data to establish their views. The topic guide is included in the supplementary materials. Vaccination coverage and attitudes formed only part of the focus groups and only the first workstream is covered in this paper but full topic guides are included for full clarity. Participants were offered an honourarium of a £50 voucher for participation in line with National Institute for Health Research guidance.^22^

### Data analysis

Focus groups and interviews were recorded and transcribed verbatim. Individual transcripts were allocated codes so they could not be identified. Thematic analysis was conducted on the transcripts using constant comparison analysis.^23^

Coding of each transcript took place by two researchers (AK and JH) independently. They met regularly to compare coding for each transcript and to form connections, resulting in themes that were included in the analysis.

### Participant and public involvement

The SIREN study runs a comprehensive engagement programme, which provides participants with regular opportunity to engage with and provide feedback on the study. These include monthly newsletters, interactive webinars, celebration events, plain language summaries of key research, a dedicated SIREN webpage and a participant experience survey. The PIP, which is cochaired by the British Society of Immunology and SIREN research team, consists of ten SIREN participants meeting every 6 weeks over a 6-month cycle. This qualitative research substudy was presented to the PIP in advance, and they formed one of the focus groups. The findings of this work will be presented at future webinars and a plain language summary will be created for the wider public.

## Results

A total of 6048 participants were still in active follow-up in the SIREN Study in March 2023 and so were included in the quantitative vaccine coverage analysis. Most participants were women (83%) and of white ethnicity (88%) and nurses made up the largest occupational group (33%) (for full demographic details see online supplemental table 2). Self-reported vaccine coverage from surveys closely matched the NIMS data.

### Influenza and COVID-19 vaccine coverage

**Figure 1:**
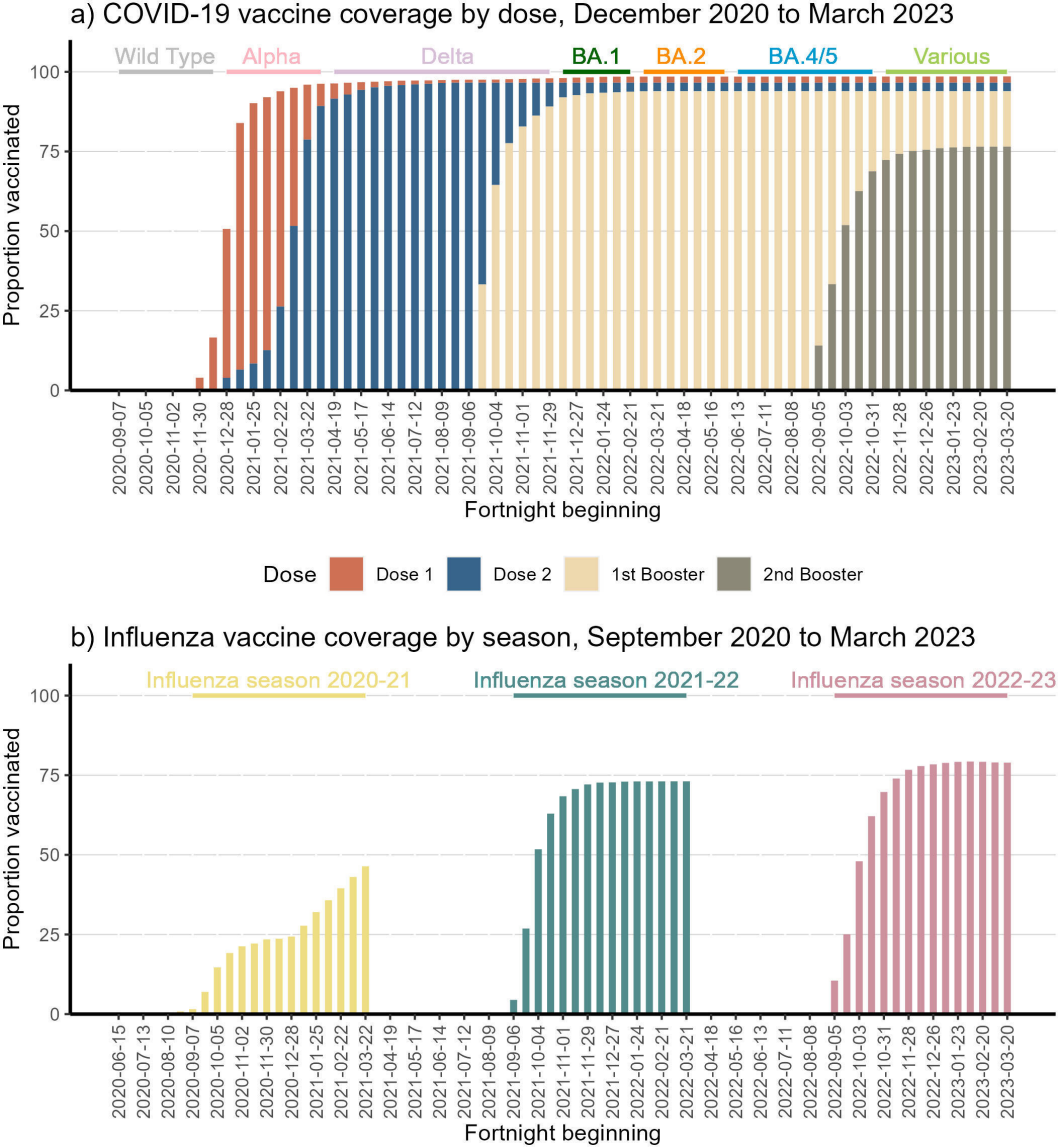
Proportion of active participants vaccinated, by dose/season and fortnight, against (a) COVID-19 and (b) influenza, December 2020 to March 2023 influenza seasons were defined as 1 September to 31 March each year.

The proportion of participants vaccinated for COVID-19 and influenza by season 2020–2021 to 2022–2023 is shown in figure 1a,b respectively. COVID-19 vaccination coverage in the SIREN cohort was initially very high with 97% receiving the primary course COVID-19 vaccination (two doses) and remained high for the first booster (94%) (figure 1a). The rate of uptake was faster for the first three vaccines than for the second booster and coverage was lower (77%).

In contrast to COVID-19 vaccine coverage, influenza coverage was lowest in 2020–2021 (46%), increasing to 73% in 2021–2022 and to 79% in 2022–2023 (figure 1b). For 2022–2023 influenza vaccination coverage was higher than COVID-19, a reversal of previous years. The rate of influenza vaccination was similar to that of COVID-19, with 62% receiving the influenza vaccine and 63% the COVID-19 vaccine within 8 weeks of roll-out in 2022–2023.

### Demographic and occupational factors associated with vaccine coverage

With the exception of the COVID-19 vaccine primary course, coverage for both influenza and COVID-19 vaccination over the previous 3 years differed by demographic and occupational factors (table 1). In general, the highest coverage was observed in older age groups, individuals of white ethnicity and participants employed as doctors, pharmacists and therapists.

**Table 1:** Number and proportion of participants vaccinated by dose/season and demographic groups

	COVID-19 primary course	COVID-19 first booster	COVID-19 second booster	Influenza season 2020–2021	Influenza season 2021–2022	Influenza season 2022–2023
Gender	N (%)	N (%)	N (%)	N (%)	N (%)	N (%)
Female	4856 (96.4)	4724 (93.8)	3812 (75.7)	2346 (46.6)	3693 (73.3)	3972 (78.9)
Male	979 (97.4)	952 (94.7)	813 (80.9)	455 (45.3)	723 (71.9)	821 (81.7)
Other	4 (66.7)	4 (66.7)	3 (50.0)	4 (66.7)	4 (66.7)	4 (66.7)
Age group (age)						
Under 35	525 (93.8)	503 (89.8)	363 (64.8)	240 (42.9)	375 (67.0)	396 (70.7)
35–44	1262 (95.8)	1212 (92.0)	900 (68.3)	608 (46.2)	977 (74.2)	999 (75.9)
45–54	2272 (97.7)	2207 (94.9)	1842 (79.2)	1103 (47.4)	1732 (74.5)	1864 (80.1)
55–64	1654 (96.5)	1633 (95.3)	1408 (82.1)	797 (46.5)	1265 (73.8)	1428 (83.3)
Over 65	126 (96.2)	125 (95.4)	115 (87.8)	57 (43.5)	71 (54.2)	110 (84.0)
Ethnic group						
White	5165 (97.0)	5040 (94.7)	4182 (78.5)	2551 (47.9)	3991 (75.0)	4297 (80.7)
Asian	355 (96.7)	343 (93.5)	249 (67.8)	145 (39.5)	224 (61.0)	269 (73.3)
Black	145 (89.0)	133 (81.6)	79 (48.5)	45 (27.6)	89 (54.6)	101 (62)
Mixed race	92 (88.5)	88 (84.6)	68 (65.4)	40 (38.5)	67 (64.4)	76 (73.1)
Other ethnic group	71 (92.2)	65 (84.4)	43 (55.8)	23 (29.9)	41 (53.2)	45 (58.4)
Prefer not to say	11 (84.6)	11 (84.6)	7 (53.8)	1 (7.7)	8 (61.5)	9 (69.2)
Occupation						
Nursing	1910 (96.6)	1858 (94.0)	1474 (74.6)	895 (45.3)	1443 (73.0)	1552 (78.5)
Administrative/executive (office based)	976 (97.1)	950 (94.5)	774 (77.0)	451 (44.9)	731 (72.7)	807 (80.3)
Other	791 (95.6)	771 (93.2)	622 (75.2)	379 (45.8)	593 (71.7)	637 (77.0)
Doctor	781 (97.5)	767 (95.8)	671 (83.8)	424 (52.9)	638 (79.7)	691 (86.3)
Healthcare assistant	349 (94.3)	338 (91.4)	249 (67.3)	159 (43.0)	229 (61.9)	271 (73.2)
Healthcare scientist	238 (96.4)	233 (94.3)	197 (79.8)	128 (51.8)	193 (78.1)	201 (81.4)
Therapists*	228 (98.3)	221 (95.3)	194 (83.6)	120 (51.7)	179 (77.2)	192 (82.8)
Pharmacist	185 (97.9)	180 (95.2)	161 (85.2)	109 (57.7)	152 (80.4)	157 (83.1)
Estates/porters/security	145 (96.7)	138 (92.0)	111 (74.0)	34 (22.7)	98 (65.3)	112 (74.7)
Midwife	122 (96.1)	115 (90.6)	88 (69.3)	56 (44.1)	72 (56.7)	91 (71.7)
Student	114 (92.7)	109 (88.6)	87 (70.7)	50 (40.7)	92 (74.8)	86 (69.9)

* Therapists includes: physiotherapist, occupational therapist and speech and language therapist.

**Figure 2:**
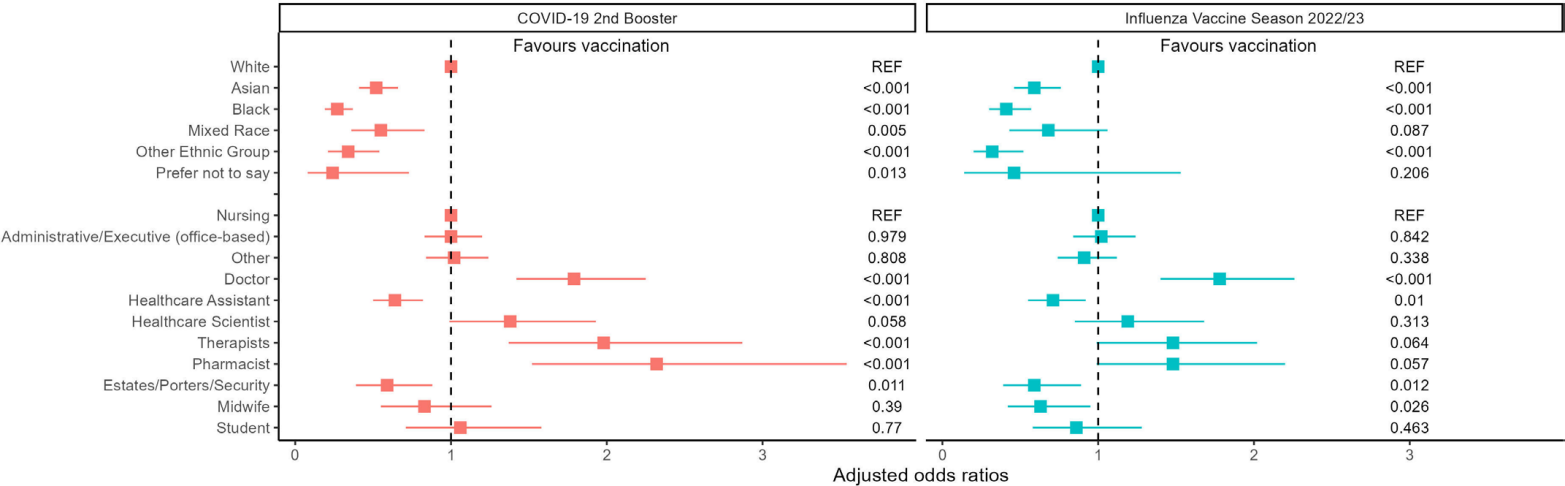
Adjusted ORs (aORs) for COVID-19 booster and Influenza 2022–2023 vaccine coverage, by ethnic group and occupation, with p values aORs calculated from a multivariable logistic regression model, including age group, gender, ethnic group and occupation group. Squares and horizontal lines represent aORs for each group and their 95% CIs. P values are shown on the right-hand side of each plot. The dashed line represents no effect (aOR=1). aORs>1 represent higher vaccine coverage and aORs<1 represent lower vaccine coverage. For ethnic group, white is the reference group. For occupation, nursing is the reference group.

In multivariable analysis, we compared vaccine coverage in the 2022–2023 season (influenza and second COVID-19 booster) by occupation and ethnicity, and controlled for age and gender, to calculate aOR. Two distinct groups emerged (figure 2). Group one had high levels of vaccine coverage for both influenza and COVID-19, which included therapists, pharmacists and doctors, and group two had low vaccine coverage for both influenza and COVID-19, which included healthcare assistants, estates staff/porters and participants in minority ethnic groups. There were no other groups where vaccine coverage was significantly higher for one vaccine than the other (figure 2; online supplemental table S1). Overall, influenza vaccine coverage was higher than COVID-19 in winter 2022–2023, with no individual group accounting for this phenomenon.

### Focus groups and structured interviews

There were five focus groups (n=21) and three semistructured interviews (n=3) conducted with participants, involving 24 participants in total. The focus groups had an average duration of 90 min and ranged from 64 to 116 min. Semistructured interviews had an average duration of 45 min and ranged from 36 to 54 min. Participant demographics were representative of the healthcare workforce with 82% women, 26% minority ethnic, covering all working age groups from across the UK (for full demographic details see online supplemental table 3. Thematic analysis of the qualitative vaccination data identified four themes which provide further insight into the vaccine coverage data (table 2).

**Table 2:** Themes identified from focus groups and semistructured interviews conducted to explore enablers and barriers for COVID-19 and influenza vaccination between 2020 and 2023 with illustrative quotes

Theme	Subthemes	Illustrative quotes
Attitudes towards vaccination changed throughout the COVID-19 pandemic	Early in the pandemic participants saw vaccines as the only way out	Early on, people saw the vaccine, or the vaccines, as the only protection, and people were scared because it was very unknown. So, yes, huge amount of people had the vaccine
	Initial uncertainty as to management of COVID-19 led to high initial uptake	COVID was one of those conditions where we had something in our armoury already, I think we would have seen the uptake of those vaccines to be less as well. But it’s because we had nothing against it, it was just more like here’s the first thing that may save your life
	Increased experience over time reduced the perception of need for the COVID-19 vaccine	…because we had a better understanding of COVID, and then you had the variants which seem to be more transmissible but less severe, then people start thinking, okay, well I’ve had vaccines, this protects me against the worst of it. COVID’s not that bad anymore, so I probably don’t need to be having vaccines.
	Low COVID-19 vaccine efficacy beliefs as the pandemic went on were associated with low vaccine uptake	The general trend I’ve seen is at first, myself included, are all very excited to get the vaccine. Because the results from the trials were so overwhelming. Generally speaking. But now when it’s getting onto four or five vaccines, everyone starts to think it’s all a bit fishy, because they’ve already had four vaccines, and then they still get infected
	Exposure to side effects from the COVID-19 vaccines led to decreasing confidence in the vaccine	Some of them have also said that the side effects of the vaccines were worse than when they had COVID Especially with the pulmonary embolism, which has been a published thing, that there are risks of clots after vaccinations, that was the biggest scare, that people died from clots all the time, and the public are aware of that, healthcare professionals more so, and the vaccination uptake has been reduced
	COVID-19 and vaccine fatigue	This year, people are a bit tired of it. There’s the feeling about, do we have to continue doing this every single year, what’s the point? Also, the risk benefit is a bit different compared to, let’s say, 18 months ago
	There was also a continuing belief in the COVID-19 vaccine	The COVID one knocks me out for 24 hours afterwards, but I suppose I’ve come to believe in it as much as anything else. It’s not miracle magic or anything like that. Its sensible science reached by doing a lot of very hard research. So, I will continue to have it all the while it’s offered.
Data-informed vaccine decisions	Desire for more data to be shared with the public	Because for a young person, what is the harm? How many people actually had cardiac problems? How much will end up in ITU? Then we want that transparency… Of all these people that unfortunately died, how many actually had underlying issues and everything else?. The research only is there for two vaccines. It’s not there for three, four, five vaccines. We don’t have the research to back up its effectiveness. And yet it’s just rolled out without that evidence.
	Good data sharing was an enabler for influenza vaccine compared with COVID-19	It ended up a lot of people had the two. And also they’d sort of said they were following really what was going on in Australia and that they’d had much higher rates of flu. So I think that was as well in everyone’s mind that the year before we’d been more socially distanced and still wearing masks and that coming into this winter obviously we hadn’t really had that. So I think people probably quite frightened, because again what was out in the press, what was out of comms coming from our own organisation. You really need to get it. flu is likely to really rebound back this year. And I think because a lot of staff normally would have flu vaccines.
Vaccine communications in healthcare settings	Limited dialogue with HCWs increased the mistrust towards the COVID-19 vaccine	I think people who are elderly like my parents, I said definitely, 100%, but I was thinking for me because I’m younger and I had COVID already. Then I couldn’t see the evidence it worked. They were saying it protects from passing to other persons, but it didn’t. We could see first-hand it didn’t… Like I said, I’m very for vaccination and everything else. I was having flu vaccine. We had every year flu vaccine I think it’s really important to protect yourself, but I said… Explanation and things… They just said, you need to have it… I was very logical… and I’m pro, but for me it wasn’t really explained as much as it should be, I think. It was just, you must, you must. Even if you ask the question… it was feeling like you are against it. It was like labelling and you can’t even ask the questions… I’m the person that needs to understand what I need to do because I have a process and I can’t just follow whatever people are doing.
	Communications throughout the pandemic did not reflect the evolving context of the COVID-19 vaccination programme	I think there needs to be more clarity about what it is, why you’re getting vaccinated, because I think it has evolved over time. Obviously, when we first got it, it was potentially to save our own life, because people were dying regardless of age, regardless of previous health conditions… And then that’s evolved to then, when you were trying to decide whether to have your children vaccinated, knowing full well at that stage that it was very unlikely to directly benefit them, so you were doing it for society as a whole. And actually, what is the purpose now?
	There was limited reach of vaccine communications	We have in our Trust got pockets of different roles that don’t use their emails a lot, and have access to computers, so then perhaps they’re not seeing all the reminders about the vaccinations and all the notifications about them, and they’re perhaps just seeing the odd poster. Looking back, a lot of our campaign is very much about the nurses, the doctors, the therapists… I know that we do a lot of flu talks, but I don't see our porter, our estate, our plumber, our electrician will go to those flu talks about… I have experience. I go to them and tell them… I can maybe talk to 20 people
	The threat of COVID-19 vaccine mandates had a negative impact on HCWs sense of autonomy	We did have one or two people who were very reluctant to be vaccinated in the first place. But when the government/hospital said that you had to have it in order to continue working, I know they changed their mind lastminute.com but for some people they ended up having a vaccine just to supposedly keep their job even if that didn’t turn out to be the case. So they were a bit miffed. And they probably haven’t had further vaccines.
The impact of the COVID-19 vaccine on the influenza vaccine and other vaccines	COVID-19 vaccines as an enabler for the influenza vaccine	I never used to have the flu vaccine… But actually, it’s that mind shift of, it’s not really about you, it’s about other people. And I think COVID brought that to the fore in that it did affect those who were vulnerable health-wise much more severely than those that weren’t.
	COVID-19 vaccine was seen as a treatment for a pandemic, whereas the influenza vaccine was seen as seasonal	I suppose you’ve got the mindset that people knew that, perhaps have always had the flu vaccination, and then just looked at the COVID as a temporary thing on top.
	Regular vaccination with COVID-19 vaccines has led to vaccine fatigue	There has been a couple of staff members who have actually not gone for their hep B immunisation because they have felt that they’d had enough needles that year.
	The availability of having both COVID-19 and influenza vaccines at the same time influenced decisions	Had I not been offered the flu and the COVID at pretty much the same time, I know in my heart of hearts that had it just been the COVID, I might have found reasons not to go, knowing that the COVID vaccine will give me a miserable 24 hours a little bit later. But because the flu vaccine was being offered as well, that was up at the level of, I know what it’s going to do to me, but I’m already having one needle stuck in, so I might as well have two and have done. I think combining the two as one session was a clincher, from my personal perspective for the COVID vaccine.

HCWs, healthcare workers.

### Theme 1: Attitudes towards vaccination changed throughout the COVID-19 pandemic

Participants described managing considerable uncertainty during the initial phase of the pandemic with vaccination seen as ‘the only way out’. The primary course of COVID-19 vaccines was perceived as providing protection to reduce an immediate threat, including unknown risks which contributed to high uptake.

People [doctors] at the beginning were in the queue because you didn’t want to end up in ITU (Site Team, FG2)

Over the course of the COVID-19 pandemic, attitudes towards the COVID-19 vaccine among participants became less positive in general, although for some, the belief in its value remained. As participants gained more experience in managing COVID-19 and saw a reduction in severity, there was a belief that the boosters were unnecessary and vaccine confidence had reduced. Increasingly, participants had contracted COVID-19 and considered it had minimal impact on them compared with the impact of COVID-19 vaccine adverse effects.

Participants also reported that they had managed patients admitted with severe adverse events of the COVID-19 vaccines, including myocarditis and thromboembolism, and mentioned that colleagues who had been caring for these patients had become disinclined to receive booster doses, particularly if they were younger. As the pandemic changed to the ‘living with COVID-19’ phase, participants had increased capacity to engage in a more active decision-making process for vaccines. They weighed up the pros and cons of vaccination when deciding whether to have further booster vaccinations, with some deciding the risk outweighed the benefit.

They’re thinking, what’s the logic? You tell me I take this vaccine and I will protect myself and protect my family and yet I still manage to get COVID? (Site Team, FG3)

### Theme 2: Data were used to inform decisions on vaccination

Participants reported the need for data transparency to increase trust in vaccines. Data including the number of COVID-19 vaccinations administered, the rate and nature of side effects, negative outcomes of vaccination and the real-world evidence of vaccine effectiveness were vital in helping them to decide whether to have COVID-19 boosters. A perceived lack of sharing of this data by those promoting vaccination within their workplace increased scepticism towards COVID-19 boosters.

We want that transparency…of all the people that unfortunately died, how many actually had underlying issues (Site Team, FG2)

In contrast, particularly before the 2022–2023 influenza season, some trusts shared data on the recent Australian influenza season demonstrating high rates of influenza seen and explaining that a particularly severe influenza season was predicted. This was a major factor in participants seeking out the influenza vaccine. Data allowed participants to perform individual risk versus benefit analyses to determine whether they would seek additional vaccines.

### Theme 3: Poor communication in healthcare settings contributed to a reduction in vaccination

Communication was a key theme raised by participants, who felt that poor communication and a lack of ability to discuss vaccination with hospital/trust management led to mistrust towards the COVID-19 vaccine. The rationale for vaccination early in the pandemic was considered to have been clearly communicated, with the need to protect oneself and others from a highly infectious and dangerous virus. However, participants described that as the pandemic developed with the perceived reduction in risk, illness severity and vaccine side effects, the rationale for vaccination was less clearly explained. Participants felt they were unable to question hospital management on the efficacy of vaccines and the rationale for boosters. Some participants felt that by asking questions, they were seen as opposing the vaccine.

Every time if you ask the question, it will be like you are against the vaccine. It wasn’t that you were against it. You just wanted to know more. (Site Team, FG2)

There were significant concerns raised regarding the potential for mandatory vaccination against COVID-19. Although this policy was never implemented, the attempt was seen as a serious threat to autonomy and participants expressed that it had put them off vaccination (including non-COVID-19 vaccines) altogether.

Factors contributing to vaccine hesitancy were discussed, and participants identified concerns about the speed of development of the COVID-19 vaccine and the impact of side effects as potential issues. Participants also discussed that reasons for vaccine hesitancy may vary by culture and ethnicity, underscoring the importance of understanding and addressing these differences for future vaccination campaigns.

There was a perception that hospital communications were primarily directed towards doctors, nurses and therapists, rather than ancillary staff and other wider healthcare professionals. Examples included trust emails with many staff members, for example, porters, not regularly having access to computers as part of their work pattern. Examples of ‘influenza talks’ to promote vaccination were given as another method of communication; however, again they were seen to miss certain occupations.

### Theme 4: The impact of the COVID-19 vaccine on the influenza vaccine and other vaccination programmes

The COVID-19 vaccination campaign had both positive and negative impacts on influenza vaccine uptake and other vaccination programmes more widely. Participants described how COVID-19 provided a reminder of the necessity and utility of vaccination and increased the awareness of the benefit not only to oneself but society more generally, which encouraged participants to take the influenza vaccine. However, there was also the negative impact of too many vaccinations. Participants described ‘vaccine fatigue’ and having multiple COVID-19 vaccines led them to be less inclined to have other vaccinations, for example, hepatitis B boosters. One method that participants described as beneficial to counteract this was the option to receive the COVID-19 booster and influenza vaccine simultaneously. The ability to have both vaccines at the same time boosted the uptake of both vaccines.

Had I not been offered the flu and COVID [vaccines] at pretty much the same time, I know… had it just been the COVID [vaccine], I might have found reasons not to go. (Participant, FG2)

The COVID-19 vaccine was associated with pandemic behaviour, whereas the influenza vaccine was seen as seasonal, more routine and habitual. Participants reported the need for data transparency, including the number of COVID-19 vaccinations administered, the rate and nature of side effects, negative outcomes of vaccination and the real-world evidence of vaccine effectiveness to help them to decide whether to have COVID-19 boosters. A perceived lack of sharing of this data increased scepticism towards COVID-19 boosters.

## Discussion

HCWs were one of the first groups to be offered the COVID-19 vaccination. Within the SIREN study, despite universally high coverage of the first three COVID-19 vaccine doses in our cohort of UK HCWs, coverage of the second booster was lower and more variable. This trend was shared with national HCW data, although coverage in SIREN has been consistently higher than observed nationally. National coverage of the second COVID-19 booster in HCW decreased from 88% (primary course) to 68% (first booster) to 42% (second booster).^7 9 24^ Seasonal influenza vaccine coverage in the SIREN cohort, meanwhile, has increased, but continues to differ by occupation, ethnic group and age. Our observed increase in SIREN contrasts with national HCW data where influenza vaccination coverage declined nationally, with 49% vaccinated in 2022–2023 compared with 61% in 2021–2022 and 77% in 2020–2021. National influenza vaccination coverage in HCWs for 2022/2023 was substantially below the target of 75% for clinical staff set via the Commissioning for Quality and Innovation (CQUIN) framework,^25^ and the lowest recorded since the introduction of the CQUIN in 2023.^24^ These persisting differences by occupation, ethnic group and age, which have been similarly reported across other HCW populations globally, are important to address.^26^,^30^

Using a mixed-methods design enabled us to explore underlying factors within HCWs probing attitudes and identifying barriers and facilitators, with four themes identified. Several relate to communication and information sharing, with a request for more open dialogue to support individual decision-making, alongside potential underserved occupational groups outside the reach of current communication campaigns. These findings have informed some possible recommendations for future COVID-19 and influenza vaccine deployment for HCWs, which may improve uptake (box 1).

Box 1 **Recommendations**

- Clear and accessible vaccine communications should be designed that reflect the scientific understanding tailored for different clinical and non-clinical groups of healthcare workers (HCWs) and shared via trusted communicators.
- Increase accessibility and reach of vaccine communications using varied modes of delivery.
- Hospital management and senior clinicians should provide the opportunity for HCWs to engage in consultation and two-way dialogue to identify and address informational needs and concerns. Communication should be collaborative and equal, promoting shared decision-making.
- Data on vaccine effectiveness and adverse events should be shared with HCWs and the positive rationale for vaccination clearly explained to aid decision-making and to build trust.
- The number of vaccination appointments should be reduced where possible, so if offering COVID-19 and influenza vaccines, they should be offered together.
- Structural barriers to vaccination should be minimised to facilitate vaccine uptake.
- There should be an avoidance of mandates to reduce mistrust and promote autonomy.
- More granular data on job roles and demographic details should be collected to identify which groups require additional support.

The lower coverage of the second COVID-19 boosters, and the themes emerging from the focus groups, underscore the importance of inclusive and sensitive communications for future vaccine roll-outs, supporting HCWs to make informed decisions. The reasons raised by participants for the significant reduction in coverage of the second COVID-19 booster compared with first booster in our cohort of HCWs in our qualitative enquiry were multifactorial, with a clear change in the participant’s risk-benefit assessment, particularly around the value of a second booster and concerns about side-effects. In discussion around facilitating ease of access to vaccines for HCWs, co-administration of COVID-19 and influenza vaccines was viewed favourably by participants as a time-efficient initiative offered at some sites.^31^ Similar themes have emerged across other studies in the UK and globally which echoed our findings that there was a reduced willingness for boosters. Concerns relating to potential long-term side effects of vaccination, a perceived lack of efficacy of the booster vaccines and that COVID-19 was no longer a high threat have led to reduced confidence in COVID-19 boosters.^32^,^36^ Increasing negative press messaging around vaccine side effects may also have influenced attitudes towards vaccination, highlighting the importance of positive messaging about vaccination.^37 38^ In this study, the influenza vaccine was more widely accepted compared with the COVID-19 vaccine due to the availability of more data on long-term side effects and effectiveness as HCWs were more familiar with it. However, fear of side effects being worse than the disease and scepticism about vaccine effectiveness have also been seen as barriers for vaccination against influenza in other studies.^3539^,^41^

A clear consensus emerged that for most HCWs, vaccination was an active decision, with individuals making their own risk assessment, and there was an appetite for transparent data sharing on benefits and potential adverse side-effects, and open channels for dialogue. In health decision-making, individuals navigate choices involving weighing the risk of consequences with the benefits of action.^42^ Risk perceptions involve incorporating information about a threat and the ability to produce, understand and use the information plays an important role in the formation and use of risk perceptions. HCWs hold a unique position for vaccination, both because they are eligible for many and have an influential role in promoting their use to patients.^43^ They are likely to be more scientifically literate, and sharing communications that reflect this literacy may increase trust in the vaccine. The proposal for mandatory COVID-19 vaccination for HCWs was viewed very negatively by participants, similar to other studies.^44 45^ Promoting evidence-based assessments for individuals by providing sufficient, accurate information to make decisions on vaccination is key.^35 39 41^

Our finding of variance in vaccine coverage between ethnic groups and occupations within HCWs is important, particularly given the increased risks of infection, morbidity and mortality over the course of the COVID-19 pandemic among some minority ethnic groups and occupations and reports of increased vaccine hesitancy among some minority ethnic groups.^46^ Understanding how and whether barriers and facilitators to vaccination among HCWs may differ by ethnicity and occupation is an important area for further research. Our focus groups identified barriers for some occupational groups that do not receive or may miss vaccine communications, with vaccine reminders often sent by email, which miss staff members who do not spend time on computers during their shifts, for example, porters, estates and HCAs. Often, posters were the only mechanism used to target these groups, and more accessible communication to reach a wider range of occupations is advised.^47^ Previously, porters and other HCWs who worked in ward settings but not directly caring for patients did not automatically qualify for the influenza vaccine (although some trusts chose to vaccinate these groups). However, this policy changed in 2022 and enhanced communication of this directed at this group may be required to improve uptake.^43 48^

The use of a mixed methods design nested within an ongoing prospective cohort study is a key strength of this study: enabling us to rapidly explore trends in our data directly with our participants to help understand possible reasons for the differences in coverage observed and inform recommendations for action. The scale of SIREN, with participants drawn from NHS sites across the UK – with participants from 71 sites contributing to this analysis, allows us to investigate national trends, and to compare different approaches to vaccination across our sites. The detailed information on our well-characterised cohort, including occupation, means we are providing new insight. National surveillance data is aggregated by ‘doctors’, ‘nurses’, ‘other clinical’ and ‘support’ staff groups. This contrasts with the more detailed occupational data available in SIREN and likely obscures heterogeneity within groups. In particular, the ‘other clinical’ group includes therapists, HCAs and estates staff/porters who had variable coverage in the SIREN data. The qualitative research was conducted by independent researchers who encouraged participants to discuss their experiences of SIREN freely. The different backgrounds of the qualitative researchers (a health psychologist and a public health registrar) enabled broader theoretical and practical considerations, which strengthened the analysis and recommendations for practice.

Key limitations from the vaccine coverage analysis include the representativeness of our cohort, with relatively small numbers in key occupational and minority ethnic groups, impacting the precision of our estimates in these groups, and the generalisability of our findings to the wider healthcare workforce. Participants who engage in research may not be representative of the workforce as a whole and may also be influenced by their experiences as a research participant. For example, SIREN participants likely had an increased awareness of the importance of influenza vaccination as we were expanding the scope of the study to include both influenza and RSV and so communications highlighted their importance, and this may have increased coverage in our cohort. For the qualitative methodology, it is important to recognise that the design – focus groups and in-depth interviews – is intended to be exploratory and although efforts were made to ensure that participants were drawn from a variety of occupations, hospital settings/regions, ethnic groups, and ages, selection bias may occur and participants from less engaged SIREN cohorts may have offered different perspectives that were not included in this study meaning there may be information bias.

## Conclusion

In conclusion, while UK HCWs are currently being offered additional booster doses of the COVID-19 vaccine alongside the influenza vaccine, we have found inequalities in the 2022–2023 deployment and identified barriers through focus groups and developed recommendations. These need to be addressed to implement a sustainable and effective seasonal vaccination programme among HCWs to reduce winter pressures.

## Supplementary material

10.1136/bmjopen-2025-113889 online supplemental file 1

10.1136/bmjopen-2025-113889 online supplemental file 2

10.1136/bmjopen-2025-113889 online supplemental file 3

## Data Availability

Data are available upon reasonable request.

## References

[1] UKHSA (2023). Vaccinations in united kingdom GOV.UK: UK health security agency. https://coronavirus.data.gov.uk/details/vaccinations.

[2] Hall VJ, Foulkes S, Saei A (2021). COVID-19 vaccine coverage in health-care workers in England and effectiveness of BNT162b2 mRNA vaccine against infection (SIREN): a prospective, multicentre, cohort study. Lancet.

[3] Watson OJ, Barnsley G, Toor J (2022). Global impact of the first year of COVID-19 vaccination: a mathematical modelling study. Lancet Infect Dis.

[4] Pople D, Monk EJM, Evans S (2022). Burden of SARS-CoV-2 infection in healthcare workers during second wave in England and impact of vaccines: prospective multicentre cohort study (SIREN) and mathematical model. BMJ.

[5] Olivera Mesa D, Hogan AB, Watson OJ (2022). Modelling the impact of vaccine hesitancy in prolonging the need for Non-Pharmaceutical Interventions to control the COVID-19 pandemic. *Commun Med (Lond*).

[6] Thorsteinsdottir B, Madsen BE (2021). Prioritizing health care workers and first responders for access to the COVID19 vaccine is not unethical, but both fair and effective - an ethical analysis. Scand J Trauma Resusc Emerg Med.

[7] UKHSA (2022). Seasonal influenza and COVID-19 vaccine uptake (frontline healthcare workers - all NHS England trusts).

[8] UKHSA (2021). Factors influencing COVID-19 vaccine uptake among minority ethnic groups.

[9] UKHSA (2022). Seasonal influenza and COVID-19 vaccine uptake amongst frontline healthcare workers (HCWS). https://www.gov.uk/government/publications.

[10] Eyre DW, Lumley SF, O’Donnell D (2020). Differential occupational risks to healthcare workers from SARS-CoV-2 observed during a prospective observational study. Elife.

[11] van der Plaat DA, Madan I, Coggon D (2022). Risks of COVID-19 by occupation in NHS workers in England. Occup Environ Med.

[12] Greenaway C, Hargreaves S, Barkati S (2020). COVID-19: Exposing and addressing health disparities among ethnic minorities and migrants. J Travel Med.

[13] Williamson EJ, Walker AJ, Bhaskaran K (2020). Factors associated with COVID-19-related death using OpenSAFELY. Nature New Biol.

[14] Public Health England (2020). Disparities in the risk and outcomes of COVID-19. https://www.gov.uk/government/publications/covid-19-review-of-disparities-in-risks-and-outcomes.

[15] Andrews N, Tessier E, Stowe J (2022). Duration of Protection against Mild and Severe Disease by Covid-19 Vaccines. N Engl J Med.

[16] Wallace S, Hall V, Charlett A (2022). Impact of prior SARS-CoV-2 infection and COVID-19 vaccination on the subsequent incidence of COVID-19: a multicentre prospective cohort study among UK healthcare workers - the SIREN (Sarscov2 Immunity & REinfection EvaluatioN) study protocol. BMJ Open.

[17] Palinkas LA, Cooper BR, Brownson RC, Colditz GA, Proctor EK (2017). Dissemination and Implementation Research in Health: Translating Science to Practice.

[18] Tong A, Sainsbury P, Craig J (2007). Consolidated criteria for reporting qualitative research (COREQ): a 32-item checklist for interviews and focus groups. Int J Qual Health Care.

[19] Palinkas LA, Mendon SJ, Hamilton AB (2019). Innovations in Mixed Methods Evaluations. Annu Rev Public Health.

[20] Gaglio B, Henton M, Barbeau A (2020). Methodological standards for qualitative and mixed methods patient centered outcomes research. BMJ.

[21] Howells A, Aquino EN, Bose D (2023). Demonstrating the learning and impact of embedding participant involvement in a pandemic research study: the experience of the SARS-CoV-2 immunity & reinfection evaluation (SIREN) study UK, 2020-2023. Res Involv Engagem.

[22] NIHR (2022). NIHR public contributor payment policy. https://www.nihr.ac.uk/documents/nihr-public-contributor-payment-policy/31626.

[23] Boeije H (2002). A Purposeful Approach to the Constant Comparative Method in the Analysis of Qualitative Interviews. Quality & Quantity.

[24] UKHSA (2022). Seasonal influenza vaccine uptake in frontline healthcare workers in England: winterseason 2022 to 2023.

[25] NHSE (2016). National CQUIN templates.

[26] Janssen C, Maillard A, Bodelet C Hesitancy towards COVID-19 Vaccination among Healthcare Workers: A Multi-Centric Survey in France. Vaccines (Basel).

[27] Kessy SJ, Wei T, Zhou Y (2023). Vaccination willingness, vaccine hesitancy, and estimated coverage of SARS-CoV-2 vaccine among healthcare workers in Tanzania: A call for action. Immun Inflamm Dis.

[28] Mohr NM, Plumb ID, Santos León E (2023). Factors associated with the decision to receive bivalent COVID-19 booster vaccination among health care personnel. Hum Vaccin Immunother.

[29] Pal S, Shekhar R, Kottewar S (2021). COVID-19 Vaccine Hesitancy and Attitude toward Booster Doses among US Healthcare Workers. Vaccines (Basel).

[30] Toth-Manikowski SM, Swirsky ES, Gandhi R (2022). COVID-19 vaccination hesitancy among health care workers, communication, and policy-making. Am J Infect Control.

[31] Lazarus R, Baos S, Cappel-Porter H (2021). Safety and immunogenicity of concomitant administration of COVID-19 vaccines (ChAdOx1 or BNT162b2) with seasonal influenza vaccines in adults in the UK (ComFluCOV): a multicentre, randomised, controlled, phase 4 trial. The Lancet.

[32] Gu F, Lin H, Chen Z (2023). Future COVID-19 Booster Vaccine Refusal in Healthcare Workers after a Massive Breakthrough Infection Wave, a Nationwide Survey-Based Study. Vaccines (Basel).

[33] Wood RM, Juanchich M, Ramirez M (2023). Promoting COVID-19 vaccine confidence through public responses to misinformation: The joint influence of message source and message content. Soc Sci Med.

[34] Tomietto M, Simonetti V, Comparcini D (2022). A large cross-sectional survey of COVID-19 vaccination willingness amongst healthcare students and professionals: Reveals generational patterns. J Adv Nurs.

[35] Pavlovic D, Sahoo P, Larson HJ (2022). Factors influencing healthcare professionals’ confidence in vaccination in Europe: a literature review. Hum Vaccin Immunother.

[36] Bell S, Clarke RM, Ismail SA (2022). COVID-19 vaccination beliefs, attitudes, and behaviours among health and social care workers in the UK: A mixed-methods study. PLoS One.

[37] Mitsikostas DD, Aravantinou-Fatorou K, Deligianni C (2021). Nocebo-Prone Behavior Associated with SARS-CoV-2 Vaccine Hesitancy in Healthcare Workers. Vaccines (Basel).

[38] Rief W (2021). Fear of Adverse Effects and COVID-19 Vaccine Hesitancy: Recommendations of the Treatment Expectation Expert Group. *JAMA Health Forum*.

[39] Smedley J, Poole J, Waclawski E (2007). Influenza immunisation: attitudes and beliefs of UK healthcare workers. Occup Environ Med.

[40] Choucair K, El Sawda J, Assaad S (2021). Knowledge, Perception, Attitudes and Behavior on Influenza Immunization and the Determinants of Vaccination. J Epidemiol Glob Health.

[41] Lorenc T, Marshall D, Wright K (2017). Seasonal influenza vaccination of healthcare workers: systematic review of qualitative evidence. BMC Health Serv Res.

[42] Ferrer R, Klein WM (2015). Risk perceptions and health behavior. Curr Opin Psychol.

[43] UKHSA (2013). The green book.

[44] Politis M, Sotiriou S, Doxani C (2023). Healthcare Workers’ Attitudes towards Mandatory COVID-19 Vaccination: A Systematic Review and Meta-Analysis. Vaccines (Basel).

[45] Rothstein MA, Parmet WE, Reiss DR (2021). Employer-Mandated Vaccination for COVID-19. Am J Public Health.

[46] Kamal A, Hodson A, Pearce JM A Rapid Systematic Review of Factors Influencing COVID-19 Vaccination Uptake in Minority Ethnic Groups in the UK. Vaccines (Basel).

[47] Sundaram N, Duckett K, Yung CF (2018). “I wouldn’t really believe statistics” – Challenges with influenza vaccine acceptance among healthcare workers in Singapore. Vaccine (Auckl).

[48] UKHSA (2022). Guidance - National Flu Immunisation Programme 2022 to 2023 Letter.

