# Supplementary material for: Understanding barriers and enablers for vaccination against COVID-19 and influenza among healthcare workers: a mixed-methods study nested within the UK SIREN cohort
Source: BMJ Open. 2025 Dec 17;15(12):e113889. doi: 10.1136/bmjopen-2025-113889 (PMC12716576; doi:10.1136/bmjopen-2025-113889)
Supplement: online supplemental file 1 [file bmjopen-15-12-s001.docx]

**Supplementary Figures**

**Supplementary Table 1: COREQ Checklist**

| **No** | **Item** | **Guide questions/description** | **Page number of manuscript, or added here** |
| --- | --- | --- | --- |
| **Domain 1: Research team and reflexivity** | | | |
| Personal Characteristics | | | |
| 1 | Interviewer/facilitator | Which author/s conducted the interview or focus group? | AK and JH both conducted all focus groups and interviews. Page 8 |
| 2 | Credentials | What were the researcher's credentials? *E.g. PhD, MD* | AK has a PhD and is a Registered Health Psychologist. JH is a medical doctor and public health registrar. |
| 3 | Occupation | What was their occupation at the time of the study? | Professor of Health Psychology, Public Health Registrar |
| 4 | Gender | Was the researcher male or female? | Female, Male |
| 5 | Experience and training | What experience or training did the researcher have? | AK and JH have advanced research methods training and previous experience of conducting qualitative research in healthcare settings. |
| Relationship with participants | | | |
| 6 | Relationship established | Was a relationship established prior to study commencement? | No prior relationship was established between the researchers and participants. |
| 7 | Participant knowledge of the interviewer | What did the participants know about the researcher? e*.g. personal goals, reasons for doing the research* | Participants knew the researchers’ professional roles and the purpose of the research. Participants were aware the researchers were independent of the central SIREN team. Page 23 |
| 8 | Interviewer characteristics | What characteristics were reported about the interviewer/facilitator? e.g. *Bias, assumptions, reasons and interests in the research topic* | None |
| **Domain 2: study design** | | | |
| Theoretical framework | | | |
| 9 | Methodological orientation and Theory | What methodological orientation was stated to underpin the study? *e.g. grounded theory, discourse analysis, ethnography, phenomenology, content analysis* | This study was underpinned by a qualitative paradigm using focus groups and interviews and thematic analysis to provide a mechanism for understanding subjective experiences, gaining insights into motivations and actions, and avoiding assumptions. Page 9 |
| Participant selection | | | |
| 10 | Sampling | How were participants selected? *e.g. purposive, convenience, consecutive, snowball* | Purposive sampling was used to recruit participants. Page 8 |
| 11 | Method of approach | How were participants approached? e*.g. face-to-face, telephone, mail, email* | Participants were approached using emails via mailing lists and newsletters. Page 8 |
| 12 | Sample size | How many participants were in the study? | There were 24 participants in the study. Page 12 |
| 13 | Non-participation | How many people refused to participate or dropped out? Reasons? | There were no participants that dropped out of the study. |
| Setting | | | |
| 14 | Setting of data collection | Where was the data collected? e*.g. home, clinic, workplace* | All data was collected online via MS Teams. Page 8 |
| 15 | Presence of non-participants | Was anyone else present besides the participants and researchers? | Only the researchers (AK and JH) were present during the interviews and focus groups with participants. |
| 16 | Description of sample | What are the important characteristics of the sample? *e.g. demographic data, date* | Participant demographics were representative of the UK healthcare workforce. Page 12 and Supplementary Table 3 |
| Data collection | | | |
| 17 | Interview guide | Were questions, prompts, guides provided by the authors? Was it pilot tested? | Interview guides were developed by authors, these were not piloted. Pages 8-9 |
| 18 | Repeat interviews | Were repeat interviews carried out? If yes, how many? | Repeat interviews were not carried out. |
| 19 | Audio/visual recording | Did the research use audio or visual recording to collect the data? | MS Teams was used to obtain an audio recording to collect the data. Where cameras were switched on, this was also captured but did not constitute data rather it resembled in-person interactions of the data collection process. Page 8 |
| 20 | Field notes | Were field notes made during and/or after the interview or focus group? | AK and JH kept individual reflective notes concurrently after conducting interviews and focus groups. |
| 21 | Duration | What was the duration of the interviews or focus group? | The focus groups had an average duration of 90 minutes and ranged from 64 to 116 minutes. Semi-structured interviews had an average duration of 45 minutes and ranged from 36 to 54 minutes. Page 12 |
| 22 | Data saturation | Was data saturation discussed? | Data saturation was discussed and reached on all themes. Data saturation was achieved when no new issues were being raised and there was repetition. |
| 23 | Transcripts returned | Were transcripts returned to participants for comment and/or correction? | Transcripts were not returned to participants for comment or correction. |
| **Domain 3: analysis and findings** | | | |
| Data analysis | | | |
| 24 | Number of data coders | How many data coders coded the data? | Two researchers (AK and JH) independently coded the data. Page 9 |
| 25 | Description of the coding tree | Did authors provide a description of the coding tree? | There is no description of the coding tree. |
| 26 | Derivation of themes | Were themes identified in advance or derived from the data? | Themes were derived from the data. Page 9 |
| 27 | Software | What software, if applicable, was used to manage the data? | No specialist qualitative software was used to manage the data |
| 28 | Participant checking | Did participants provide feedback on the findings? | Participants did not provide feedback on the findings as this information was not provided to them. |
| Reporting | | | |
| 29 | Quotations presented | Were participant quotations presented to illustrate the themes / findings? Was each quotation identified? e*.g. participant number* | From pages 15-19, participant quotations have been presented to illustrate the theme and findings, and a participant identifier has been included alongside each quote. In addition, Table 2 includes participant quotes to illustrate each theme. |
| 30 | Data and findings consistent | Was there consistency between the data presented and the findings? | The study findings have been presented in a clear, consistent manner to reflect the data that have been collected. All findings were derived from the data and all themes are supported with illustrative quotes. Pages 12-19. |
| 31 | Clarity of major themes | Were major themes clearly presented in the findings? | Table 2 presents a succinct summary of all the major themes presented in the findings - pages 12-15. In addition, each theme is presented using separate headings - pages 15-19. |
| 32 | Clarity of minor themes | Is there a description of diverse cases or discussion of minor themes? | There was no divergence in themes or participant experiences and participants experiences have been captured and reported within the major themes. |

**Supplementary Table 2: Number and proportion of participants included in the quantitative analysis by demographic group (N=6,048)**

| **Gender** | **Number (%)** |
| --- | --- |
| Female | 5037 (83.3) |
| Male | 1005 (16.6) |
| Other | 6 (0.1) |
| **Age Group** |  |
| Under 35 | 560 (9.3) |
| 35 to 44 | 1317 (21.8) |
| 45 to 54 | 2326 (38.5) |
| 55 to 64 | 1714 (28.3) |
| Over 65 | 131 (2.2) |
| **Ethnic Group** |  |
| White | 5324 (88.0) |
| Asian | 367 (6.1) |
| Black | 163 (2.7) |
| Mixed race | 104 (1.7) |
| Other ethnic group | 77 (1.3) |
| Prefer not to say | 13 (0.2) |
| **Occupation** |  |
| Nursing | 1977 (32.7) |
| Administrative/Executive (office-based) | 1005 (16.6) |
| Other | 827 (13.7) |
| Doctor | 801 (13.2) |
| Healthcare Assistant | 370 (6.1) |
| Healthcare Scientist | 247 (4.1) |
| Therapists^a^ | 232 (3.8) |
| Pharmacist | 189 (3.1) |
| Student | 150 (2.5) |
| Estates/Porters/Security | 127 (2.1) |
| Midwife | 123 (2.0) |

^a^ Therapists includes: Physiotherapist, Occupational Therapist, and Speech & Language Therapist

**Supplementary Table 3: Number and proportion of participants included in the qualitative analysis by demographic group (N=24)**

|  | **Count** | **Percentage** |
| --- | --- | --- |
| **Age^[[1]](#footnote-1)^** |  |  |
| 25-34 | 2 | 8.3% |
| 35-44 | 5 | 20.8% |
| 45-54 | 11 | 45.8% |
| 55 or older | 6 | 25.0% |
| **Gender^[[2]](#footnote-2)^** |  |  |
| Female | 20 | 83.3% |
| Male | 4 | 16.7% |
| **Ethnicity^[[3]](#footnote-3)^** |  |  |
| Asian | 5 | 20.8% |
| Black | 1 | 4.2% |
| Mixed Race | 1 | 4.2% |
| White | 17 | 70.8% |
| **Reports long-standing illness, disability or infirmity^[[4]](#footnote-4)^** |  |  |
| Yes | 5 | 20.8% |
| No | 19 | 79.2% |
| **Region^[[5]](#footnote-5)^** |  |  |
| East of England | 5 | 20.8% |
| London | 5 | 20.8% |
| Northern Ireland | 1 | 4.2% |
| North West | 3 | 12.5% |
| Scotland | 3 | 12.5% |
| South East | 2 | 8.3% |
| Wales | 1 | 4.2% |
| West Midlands | 3 | 12.5% |
| Yorkshire and the Humber | 1 | 4.2% |

**Supplementary Table 4 STROBE Checklist**

|  | **Item No** | **Recommendation** | **Page  No** |
| --- | --- | --- | --- |
| **Title and abstract** | 1 | (*a*) Indicate the study’s design with a commonly used term in the title or the abstract | 1 |
|  |  | (*b*) Provide in the abstract an informative and balanced summary of what was done and what was found | 1 |
| **Introduction** | | | |
| Background/rationale | 2 | Explain the scientific background and rationale for the investigation being reported | 4 |
| Objectives | 3 | State specific objectives, including any prespecified hypotheses | 4 |
| **Methods** | | | |
| Study design | 4 | Present key elements of study design early in the paper | 5 |
| Setting | 5 | Describe the setting, locations, and relevant dates, including periods of recruitment, exposure, follow-up, and data collection | 5 |
| Participants | 6 | (*a*) *Cohort study*—Give the eligibility criteria, and the sources and methods of selection of participants. Describe methods of follow-up  *Case-control study*—Give the eligibility criteria, and the sources and methods of case ascertainment and control selection. Give the rationale for the choice of cases and controls  *Cross-sectional study*—Give the eligibility criteria, and the sources and methods of selection of participants | 5 |
|  |  | (*b*) *Cohort study*—For matched studies, give matching criteria and number of exposed and unexposed  *Case-control study*—For matched studies, give matching criteria and the number of controls per case | 5 |
| Variables | 7 | Clearly define all outcomes, exposures, predictors, potential confounders, and effect modifiers. Give diagnostic criteria, if applicable | 5 |
| Data sources/ measurement | 8* | For each variable of interest, give sources of data and details of methods of assessment (measurement). Describe comparability of assessment methods if there is more than one group | 5 |
| Bias | 9 | Describe any efforts to address potential sources of bias | 6 |
| Study size | 10 | Explain how the study size was arrived at | 6 |
| Quantitative variables | 11 | Explain how quantitative variables were handled in the analyses. If applicable, describe which groupings were chosen and why | 5 |
| Statistical methods | 12 | (*a*) Describe all statistical methods, including those used to control for confounding | 6 |
|  |  | (*b*) Describe any methods used to examine subgroups and interactions | 6 |
|  |  | (*c*) Explain how missing data were addressed | 6 |
|  |  | (*d*) *Cohort study*—If applicable, explain how loss to follow-up was addressed  *Case-control study*—If applicable, explain how matching of cases and controls was addressed  *Cross-sectional study*—If applicable, describe analytical methods taking account of sampling strategy | N/A |
|  |  | (*e*) Describe any sensitivity analyses | N/A |

| **Results** | | | |
| --- | --- | --- | --- |
| Participants | 13* | (a) Report numbers of individuals at each stage of study—eg numbers potentially eligible, examined for eligibility, confirmed eligible, included in the study, completing follow-up, and analysed | 8 |
|  |  | (b) Give reasons for non-participation at each stage | 8 |
|  |  | (c) Consider use of a flow diagram | N/A |
| Descriptive data | 14* | (a) Give characteristics of study participants (eg demographic, clinical, social) and information on exposures and potential confounders | 10 |
|  |  | (b) Indicate number of participants with missing data for each variable of interest | 10 |
|  |  | (c) *Cohort study*—Summarise follow-up time (eg, average and total amount) | 10 |
| Outcome data | 15* | *Cohort study*—Report numbers of outcome events or summary measures over time | 10 |
|  |  | *Case-control study—*Report numbers in each exposure category, or summary measures of exposure |  |
|  |  | *Cross-sectional study—*Report numbers of outcome events or summary measures |  |
| Main results | 16 | (*a*) Give unadjusted estimates and, if applicable, confounder-adjusted estimates and their precision (eg, 95% confidence interval). Make clear which confounders were adjusted for and why they were included | 18 |
|  |  | (*b*) Report category boundaries when continuous variables were categorized |  |
|  |  | (*c*) If relevant, consider translating estimates of relative risk into absolute risk for a meaningful time period |  |
| Other analyses | 17 | Report other analyses done—eg analyses of subgroups and interactions, and sensitivity analyses | 11 |
| **Discussion** | | | |
| Key results | 18 | Summarise key results with reference to study objectives | 18 |
| Limitations | 19 | Discuss limitations of the study, taking into account sources of potential bias or imprecision. Discuss both direction and magnitude of any potential bias | 21 |
| Interpretation | 20 | Give a cautious overall interpretation of results considering objectives, limitations, multiplicity of analyses, results from similar studies, and other relevant evidence | 21 |
| Generalisability | 21 | Discuss the generalisability (external validity) of the study results | 21 |
| **Other information** | | | |
| Funding | 22 | Give the source of funding and the role of the funders for the present study and, if applicable, for the original study on which the present article is based | 22 |

*Give information separately for cases and controls in case-control studies and, if applicable, for exposed and unexposed groups in cohort and cross-sectional studies.

**Note:** An Explanation and Elaboration article discusses each checklist item and gives methodological background and published examples of transparent reporting. The STROBE checklist is best used in conjunction with this article (freely available on the Web sites of PLoS Medicine at http://www.plosmedicine.org/, Annals of Internal Medicine at http://www.annals.org/, and Epidemiology at http://www.epidem.com/). Information on the STROBE Initiative is available at www.strobe-statement.org.

1. Participants were asked to select from: 18-24; 25-34; 35-44; 45-54; 55 or older; prefer not to say [↑](#footnote-ref-1)
2. Participants were asked to select from: woman; man; non-binary; prefer not to say; other [open text box] [↑](#footnote-ref-2)
3. Participants were asked to select from: Asian; Black; Mixed race; White; prefer not to say; other [open text box] [↑](#footnote-ref-3)
4. Participants were asked to select from: yes; no; prefer not to say

   ^5^ Participants were asked to select where they work from: East Midlands; East of England; London; North East; North West; Northern Ireland; Scotland; South East; South West; Wales; West Midlands; Yorkshire and the Humber [↑](#footnote-ref-4)
5. Participants were asked to select where they work from: East Midlands; East of England; London; North East; North West; Northern Ireland; Scotland; South East; South West; Wales; West Midlands; Yorkshire and the Humber [↑](#footnote-ref-5)
