# Supplementary material for: Understanding barriers and enablers for vaccination against COVID-19 and influenza among healthcare workers: a mixed-methods study nested within the UK SIREN cohort
Source: BMJ Open. 2025 Dec 17;15(12):e113889. doi: 10.1136/bmjopen-2025-113889 (PMC12716576; doi:10.1136/bmjopen-2025-113889)
Supplement: online supplemental file 2 [file bmjopen-15-12-s002.pdf]

SIREN

SARS-CoV2 Immunity & Reinfection Evaluation

COVID-19 SIREN Follow-Up Questionnaire

sha1 sha1Id

Welcome

Thank you for your continued participation in the study, your contribution is critical for informing the UK's response to COVID-19. We will ask you a few short questions about how you have been between {daterange}.

Your health over the past 2 weeks

Did you have a positive COVID-19 test?
Select all that apply

- ☐ No
- ☐ Yes - lateral flow test (LFT)
- ☐ Yes - PCR

Please specify the earliest date (between {daterange}) you tested positive by lateral flow test:

Please specify the earliest date (between {daterange}) you tested positive by PCR:

Did you test positive for another respiratory pathogen?

- ◯ Yes
- ◯ No

Select all that apply

- ☐ Influenza
- ☐ Respiratory Syncytial Virus (RSV)
- ☐ Non-COVID-19 coronavirus
- ☐ Parainfluenza
- ☐ Adenovirus
- ☐ Human Metapneumovirus
- ☐ Rhinovirus/Enterovirus
- ☐ Bordetella pertussis
- ☐ Chlamydia pneumoniae
- ☐ Mycoplasma pneumoniae
- ☐ Other respiratory pathogen

Please specify

Select all that apply

- ☐ Influenza A
- ☐ Influenza B
- ☐ Influenza Unsure

Please specify the earliest date (between {daterange}) you tested positive for **Influenza**:

Please specify the earliest date (between {daterange}) you tested positive for **Respiratory Syncytial Virus (RSV):**

Please specify the earliest date (between {daterange}) you tested positive for **Non-COVID-19 coronavirus**:

Please specify the earliest date (between {daterange}) you tested positive for **Parainfluenza**:

Please specify the earliest date (between {daterange}) you tested positive for **Adenovirus:**

Please specify the earliest date (between {daterange}) you tested positive for **Human Metapneumovirus:**

Please specify the earliest date (between {daterange}) you tested positive for **Rhinovirus/ Enterovirus:**

Please specify the earliest date (between {daterange}) you tested positive for **Bordetella pertussis:**

Please specify the earliest date (between {daterange}) you tested positive for **Chlamydia pneumoniae:**

Please specify the earliest date (between {daterange}) you tested positive for **Mycoplasma pneumoniae:**

Please specify the earliest date (between {daterange}) you tested positive for **Other respiratory pathogen**:

Between **{daterange}**, have you started to experience any new symptoms in the following list?
Tick all that apply

- ☐ A new, continuous cough (coughing a lot for more than an hour, or 3 or more coughing episodes in 24 hours)
- ☐ Fever or high temperature (meaning you feel hot to touch on your chest or back)
- ☐ Shortness of breath
- ☐ Sore throat
- ☐ Runny nose
- ☐ Headache
- ☐ Muscle aches
- ☐ Altered sense of smell
- ☐ Altered sense of taste
- ☐ Extreme fatigue
- ☐ Diarrhoea
- ☐ Nausea or Vomiting
- ☐ Small, itchy red patches, on fingers or toes
- ☐ Rash
- ☐ Swollen glands

- ☐ No, I have not got any new symptoms

When did these symptoms start?

Approximate dates are fine. Please use the calendar or enter in DD/MM/YYYY, e.g. 21/01/2020.
If you had more than one symptom, enter the date the first symptom started

Approximately how long did these symptoms last?

If you had more than one symptom, start counting when the first symptom appeared to when the last symptom ended.

- 1 day
- 2 days
- 3 days
- 4 days
- 5 days
- 6 days
- 7 to 14 days (1-2 weeks)
- My symptoms still have not resolved
- I am not sure

In the last 2 weeks have you taken any days off work (sick leave) due to respiratory symptoms?

- Yes
- No

Please select number of days

- 1 day
- 2 days
- 3 days
- 4 days
- 5 days
- 6 days
- 7 days
- 8 days
- 9 days
- 10 days
- 11 days
- 12 days
- 13 days
- 14 days

During this period did you seek medical care at hospital for these symptoms?

- Yes, and COVID-19 was confirmed
- Yes, and influenza was confirmed
- Yes, and another respiratory pathogen was confirmed
- Yes, but **no** respiratory pathogen confirmed
- No

During this period did you did you receive any treatment for COVID-19?

- ○ Yes
- ○ No

- ☐ Nirmatrelvir and ritonavir (Paxlovid)
- ☐ Sotrovimab (Xevudy)
- ☐ Remdesivir (Veklury)
- ☐ Molnupiravir (Lagevrio)
- ☐ Dexamethasone
- ☐ Casirivimab / imdevimab (Ronapreve)
- ☐ Tocilizumab
- ☐ Sarilumab
- ☐ Baricitinib
- ☐ Convalescent plasma
- ☐ Palivizumab
- ☐ None of the above

Please specify the earliest date you received treatment:

[ ]

Your exposures to COVID-19 at work, at home and in the community

Thinking about the period {daterange}:

During this period, how often did your job require you to be in close proximity to a suspected or confirmed COVID-19 patients?

- Every shift
- One shift a week or more, but not every shift
- One shift a month or more, but not every week
- One or two shifts in the last 2 weeks
- Never

Between {daterange} have you had contact with a suspected or confirmed case of COVID-19 **at work**?
Tick all that apply

- Yes - with a colleague
- Yes - with a patient
- Yes - with bodily fluids
- No

When you were in contact with patients with COVID-19 (suspected or confirmed, but while they were likely to be infectious) did you wear appropriate PPE?
Contact means within 2 meters, face-to-face or contact with bodily fluids. Appropriate PPE refers to wearing PPE in line with your organisational guidelines at the time.

- Always
- Most of the time
- Sometimes
- Never
- Not applicable / PPE not required for encounter(s)

Have you had any contact with suspected or confirmed COVID-19 cases without the PPE spcified by your workplace?
Tick all that apply

- Yes – Experienced a breach / contamination whilst wearing PPE
- Yes - Unexpected encounter with a patient or bodily fluids whilst not wearing PPE
- No – I have not had any contact with COVID-19 without PPE specified by your workplace

Between {daterange} do you believe you were in contact with someone with COVID-19 **at home**?
Include shared house occupants and visitors, if they were living in your household

Tick all that apply

- Yes, suspected case
- Yes, confirmed case (positive PCR or LFT)
- No
- I don't know / I can't remember

Between {daterange} do you believe you were in contact with someone with COVID-19 **in any other setting**?
Include visiting friends and family, pubs, restaurants, place of worship and shopping

Tick all that apply

- ☐ Yes, suspected case
- ☐ Yes, confirmed case (positive PCR or LFT)
- ☐ No
- ☐ I don't know / I can't remember

When did the person living in your household become unwell with COVID-19 like illness?
If more than one person got ill in the past 2 weeks, give the date the first person became unwell.
Please use the calendar or enter as DD/MM/YYYY, e.g. 21/01/2020.

During this period, did any household members have to go to hospital due to COVID-19 related symptoms?

- ○ Yes – COVID-19 was suspected/confirmed
- ○ Yes – COVID-19 was not confirmed
- ○ No
- ○ I am not sure

Between {daterange} were you informed through contact tracing that you had been in contact with a confirmed COVID-19 case?

- ○ Yes
- ○ No
- ○ I don't know / I can't remember

When were you informed, through contact tracing, that you had been in contact with a confirmed COVID-19 case?
Approximate dates are fine. Please use the calendar or enter as DD/MM/YYYY, e.g. 21/01/2020.

COVID-19 Vaccines and Prophylaxis treatment

Have you had a COVID-19 vaccination, including a booster?

Include
- Boost dose(s) as the third, fourth or fifth dose
- Vaccines from abroad

Exclude
- Vaccines as part of a trial (there is a question on trials later)

- ◯ Yes - I have had a vaccine dose I have not yet reported
- ◯ Yes – I already reported my vaccine dose(s) on a previous questionnaire
- ◯ No - I have not been vaccinated against COVID-19

* If you receive a COVID vaccine please note the manufacturer, date and batch number

Which vaccine dose(s) are you reporting?

- ☐ First dose
- ☐ Second dose
- ☐ Third dose (Booster)
- ☐ Fourth dose (Booster)
- ☐ Fifth dose (Booster)

Date of **first** dose:

[ ]

What is the name/manufacturer of your **first** dose?

If you do not know the name, please enter 'don't know'

- ◯ Pfizer-BioNTech
- ◯ Oxford-AstraZeneca
- ◯ Moderna (Spikevax)
- ◯ Janssen
- ◯ Nuvaxoid (Novavax)
- ◯ Other

Other

If unsure, please enter 'don't know'

[ ]

What is the batch number for your **first** dose?

If unsure, please enter 'don't know'

[ ]

Date of **second** dose:

[ ]

What is the name/manufacturer of your **second** dose?
If you do not know the name, please enter 'don't know'

- ○ Pfizer-BioNTech
- ○ Oxford-AstraZeneca
- ○ Moderna (Spikevax)
- ○ Janssen
- ○ Nuvaxoid (Novavax)
- ○ Other

Other
If unsure, please enter 'don't know'

What is the batch number for your **second** dose?
If unsure, please enter 'don't know'

Date of **third (booster)** dose:

What is the name/manufacturer of your **third (booster)** dose?
If you do not know the name, please enter 'don't know'

- ○ Pfizer-BioNTech
- ○ Oxford-AstraZeneca
- ○ Moderna (Spikevax)
- ○ Janssen
- ○ Nuvaxoid (Novavax)
- ○ Other

Other
If unsure, please enter 'don't know'

What is the batch number for your **third (booster)** dose?
If unsure, please enter 'don't know'

Date of **fourth (booster)** dose:

What is the name/manufacturer of your **fourth (booster)** dose?
If you do not know the name, please enter 'don't know'

- ○ Pfizer-BioNTech
- ○ Oxford-AstraZeneca
- ○ Moderna (Spikevax)
- ○ Janssen
- ○ Nuvaxoid (Novavax)
- ○ Other

Other
If unsure, please enter 'don't know'

[ ]

What is the batch number for your **fourth (booster)** dose?
If unsure, please enter 'don't know'

[ ]

Date of **fifth (booster)** dose:

[ ]

What is the name/manufacturer of your **fifth(booster)** dose?
If you do not know the name, please enter 'don't know'

- ○ Pfizer-BioNTech
- ○ Oxford-AstraZeneca
- ○ Moderna (Spikevax)
- ○ Janssen
- ○ Nuvaxoid (Novavax)
- ○ Other

Other
If unsure, please enter 'don't know'

[ ]

What is the batch number for your **fifth(booster)** dose?
If unsure, please enter 'don't know'

[ ]

Have you had a seasonal flu vaccine for 22/23 season?

- ○ Yes - I have had a vaccine I have not yet reported
- ○ Yes - already reported on a previous questionnaire
- ○ No - I have not had a seasonal flu vaccine for 22/23 season

If yes, what date did you have your flu vaccination?

[ ]

What is the name and manufacturer?

- ◯ Influvac sub-unit Tetra, Quadrivalent Influenza Vaccine, egg-grown (QIVe) (Viatris)
- ◯ Cell-based Quadrivalent Influenza Vaccine (QIVc) (Seqirus)
- ◯ Supemtek, recombinant Quadrivalent Influenza Vaccine (QIVr) (Sanofi Pasteur)
- ◯ Adjuvanted Quadrivalent Influenza Vaccine (aQIV) (Seqirus)
- ◯ Don’t know

Did you receive the flu vaccine at work?

- ◯ Yes
- ◯ No

Have you enrolled in any COVID-19 vaccine or prophylaxis treatment trials that you have not yet reported to us?

Prophylaxis treatment refers to medication you take when you are well to try to prevent illness. Please only tick “Yes” if you have participated in a study where you were given prophylaxis treatment or a vaccine. Studies only involving testing should not be considered.

- ◯ Yes - I have enrolled in a new trial
- ◯ Yes - I have enrolled in a new prophylaxis treatment trial
- ◯ No - I am part of a trial I have already reported
- ◯ No – I am not enrolled in any trials

If yes, please provide the name of the trial

Travel Information

Between {daterange}, did you travel abroad?

- ◯ Yes
- ◯ No
- ◯ I don't know / I can't remember

If yes, please only put the name of the country

Country 1 ____________________

Country 2 ____________________

Country 3 ____________________

Country 4 ____________________

Country 5 ____________________
