# Supplementary material for: Understanding barriers and enablers for vaccination against COVID-19 and influenza among healthcare workers: a mixed-methods study nested within the UK SIREN cohort
Source: BMJ Open. 2025 Dec 17;15(12):e113889. doi: 10.1136/bmjopen-2025-113889 (PMC12716576; doi:10.1136/bmjopen-2025-113889)
Supplement: online supplemental file 3 [file bmjopen-15-12-s003.docx]

**SIREN IMPACT EVALUATION SUBSTUDY TOPIC GUIDE**

**Workstream 1**

**Opening questions**

1. What has been your involvement in the SIREN study?

**The next set of questions are about the impact of the SIREN study on staff experiences**

1. What, if anything, have been the positive aspects of the SIREN study on staff?
2. What, if anything, have been the negative aspects of the SIREN study on staff?
3. What has been the impact of regular testing within the SIREN study?
   1. *Has regular testing helped staff in any way?*
   2. *Has regular testing caused any problems for staff?*
   3. *What is it like getting a positive test in this context?*
   4. *Do test results inform whether or not people will take time off work?*
   5. *Is the SIREN study a barrier or facilitator for testing?*
4. Has the Covid-19 pandemic contributed to any changes in the way staff take time off work?
5. Has there been a change in staff attitudes towards regular vaccinations during the SIREN study?
   1. *If yes, why? If not, why not?*
   2. *Is this the case for all vaccines or does it vary, e.g., for COVID-19 boosters vs flu jabs?*
6. Recent data, suggests vaccination uptake is lower than it was at the earlier stages of the pandemic. Why do think there has been this change to vaccination uptake?
   1. *Is this the case for all vaccines or does it vary, e.g., for COVID-19 boosters vs flu jabs?*
   2. *Is it possible the SIREN study contributed to changes in vaccine coverage?*
   3. *Is the SIREN study a barrier or a facilitator for staff vaccine uptake?*
7. In your experience, does SIREN data affect local decision-making?
   1. *Have you experienced any positive changes to local hospital policy or practice as a result of the SIREN study?*
   2. *Have you experienced any negative changes to local hospital or practice as a result of the SIREN study?*
8. Are the results of the SIREN study shared with you?
   1. *If yes, has this been helpful?*
   2. *If not, would it be helpful to have this information?*
9. Can real-time data from SIREN assist with upcoming winter pressures or any future needs?
10. Is there anything else the SIREN study could include or change to improve staff experiences or is there anything else that would be helpful to know that hasn’t already been discussed?

**Workstream 2**

**Overview:**

- Participant retention and sample return are key factors for SIREN study success (and future studies like it).
- SIREN is a decentralised study involving 135 NHS organisations across the UK. Each site was responsible for recruiting participants, collecting participant samples and sharing key updates with participants on behalf of the central SIREN team.
- SIREN is reliant on its sites for study success.
- Over the course of the SIREN study there has been variability in participant retention by site, sample and questionnaire return, and engagement with the central SIREN team.
- We will run 2x focus groups with SIREN site teams to explore barrier and levers to SIREN sites participating in the study, to provide learning for future studies.
- Focus group questions will be themed around approaches to study set-up, participant recruitment, running the study and participant retention – and the challenges and factors for success associated with these.

**Participants:**

- 6 – 8 individuals per focus group.
- Participants will ideally be Research Nurses or research team members involved in the running of the SIREN study at sites.

**Participant selection metrics:**

- Participant retention (based on the percentage of participants retained in year 1 or year 1 and year 2 for sites who extended).
- Sample return rate (based on PCR and serology return rates).
- Engagement with the central SIREN study team (feedback from SIREN regional leads).

**Questions:**

**Opening question**

1. What has been your involvement in the SIREN study?

**Study set-up**

*The next set of questions will focus on your experience of setting up SIREN.*

1. Could you talk us through setting up the SIREN study at your site?
2. Any particular challenges to setting up the study at your site?
3. Any particular facilitators to setting up the study at your site?

*Prompt if needed: What resources were needed, who made up the SIREN study team, where were SIREN appointments held, working with lab colleagues.*

**Participant recruitment**

*The next set of questions are about recruiting participants at your site.*

1. Any recruitment approaches that worked well? Why?
2. Any particular challenges to recruitment?
3. Any particular facilitators to recruitment?

*Prompt if needed: please share any site-based activities you undertook to encourage participant recruitment, or any materials provided by UKHSA.*

**Running the study**

*The next set of questions are about running the study at your site.*

1. How did you find running the SIREN study at your site?
2. Did you have the resources that you needed? E.g. staff, physical space, funding, relationship with labs
3. What were factors that helped the SIREN study run smoothly?
4. What challenges did you face participating in the SIREN study?
5. Was engaging with any specific group challenging when running the SIREN study?
6. Did you engage with the UKHSA SIREN study team while running the SIREN study at your site?
7. If so, what worked well? Why?
8. What could have been improved? Why?
9. What was your experience of retaining participants in the study?
10. Do you know why people dropped out of the study?
11. Do you know why people stayed in the study?
12. Is there anything that would have made it easier to retain participants?

*Prompt if needed: Would more of the following have helped? Incentives e.g. prizes, more events put on by UKHSA, recognition e.g. certificates, badges. Would less frequent testing of participants helped?*

**Closing question (if time allows)**

1. Is there anything else regarding recruitment or the running of SIREN at your study site that would be useful for us to know?
2. Is there anything you would like to share that can help improve the way SIREN or similar studies run in the future?
