# Supplementary material for: Understanding barriers and enablers for vaccination against COVID-19 and influenza among healthcare workers: a mixed-methods study nested within the UK SIREN cohort
Source: BMJ Open. 2025 Dec 17;15(12):e113889. doi: 10.1136/bmjopen-2025-113889 (PMC12716576; doi:10.1136/bmjopen-2025-113889)
Supplement: Supplementary file 4 [file bmjopen-2025-113889.reviewer_comments.pdf]

PEER REVIEW HISTORY

BMJ Open publishes all reviews undertaken for accepted manuscripts. Reviewers are asked to complete a checklist review form and are provided with free text boxes to elaborate on their assessment. These free text comments are reproduced below.

ARTICLE DETAILS

Title (Provisional)

Understanding barriers and enablers for vaccination against COVID-19 and influenza amongst healthcare workers a mixed-methods study nested within the UK SIREN cohort

Authors

Sparkes, Dominic; Munro, Katie; Kamal, Atiya; Haywood, Jack; Howells, Anna; Foulkes, Sarah; Russell, Sophie; Platt, Naomi; Broad, Jonathan; Brown, Colin S; Hopkins, Susan; Islam, Jasmin; Hall, Victoria; Study Team, SIREN

VERSION 1 - REVIEW

**Reviewer**	**1**
**Name**	**Anastasia Saade**
**Affiliation**	**Université de Rennes, IRSET**
**Date**	**13-Jan-2025**
**COI**	**Pfizer**

I made a conference for pfizer but nothing to do about COVID vaccination. It was about vaccination of immunocompromised patients in general.

Thank you for the opportunity to review this manuscript. This topic is of interest and indeed few studies addressed the attitude of HCWs towards influenza and COVID booster vaccination in the COVID post-pandemic era.
The strength of this work is clearly the sample size with interesting data on vaccination coverage kinetics from 2020 to 2023.
We expect new data of vaccine hesitancy provided the very big number of participants. However, this paper does not provide with something original concerning the quantitative part of this study. The qualitative data, which are much appreciate nowadays, despite its small sample size provides very interesting data, highlighting the lack of information on vaccine risks and benefits.

Major comments :
The manuscript could probably be written in a different manner to make it more fluent.
You state that your study adds : identification of HCWs groups with high and low vaccine

coverage. This question was already reported in a couple of papers in the literature showing that doctors much often receive influenza annual vaccination compared to nurses or nurse assistants.
Methods : Do you use a multivariable analysis ? or a multivariate analysis ? Please state the software used for your statistical analysis.
Would cluster analysis be more stringent ?
Wasn't COVID vaccination compulsory for HCWs ? if this was the case please state it in the introduction.
Your quantitative analysis does not provide data on barriers and facilitators but rather the qualitative data alone. Please temper these sentences in your manuscript.
Qualitative data do not distinguish between HCWs categories ? why didn't you provide with this data ?

Minor comments :
Introduction
Line 45 : add a comma after "infections and mortality"

Methods
Page 6 Line 22 : "A mixed-methods study nested within the SIREN multicentre prospective cohort study of HCWs across the UK" please add a verb to the sentence
Page 6 Line 48 :"COVID-19 and influenza vaccine data (date and type) was obtained" ==> were obtained ?
Page 7 line 20 : "year, in line with dates HCWs can receive each vaccine)" : I do not understand that sentence.
Page 7 : I am wondering whether this paragraph would better fit in the study design section
"Vaccine coverage (the proportion of the cohort vaccinated) was calculated for each of the four
COVID-19 vaccines, and for each influenza season (defined as 1st September until 31st March each
year, in line with dates HCWs can receive each vaccine) between September 2020 and March 2023
(3 seasons). For COVID-19 boosters, the 1st booster season was defined as 01/09/2021-31/03/2022,
and the 2nd booster as 01/09/2022-31/03/2023."

References :
Please discuss
Mohr NM, Plumb ID, Santos León E, Harland KK, Krishnadasan A, Nandi U, et al. Factors associated with the decision to receive bivalent COVID-19 booster vaccination among health care personnel. Hum Vaccines Immunother. 2023;19:2284471.
SteelFisher GK, Findling MG, Caporello HL, McGowan E, Espino L, Sutton J. Divergent

Attitudes Toward COVID-19 Vaccine vs Influenza Vaccine. JAMA Netw Open.2023;6:e2349881.
Kessy SJ, Wei T, Zhou Y, Zhang W-X, Alwy Al-Beity FM, Zhang S-S, et al. Vaccination willingness, vaccine hesitancy, and estimated coverage of SARS-CoV-2 vaccine among healthcare workers in Tanzania: A call for action. Immun Inflamm Dis. 2023;11:e1126.
Choi Y, Park S, Lee J, Kim Y, Kim BJ, Lin L, et al. Who gets COVID-19 booster vaccination? Trust in public health institutions and promotion strategies post-pandemic in the Republic of Korea. Vaccine. 2023;41:7560-72.
Durando P, Alicino C, Dini G, Barberis I, Bagnasco AM, Iudici R, et al. Determinants of adherence to seasonal influenza vaccination among healthcare workers from an Italian region: results from a cross-sectional study. BMJ Open. 2016;6:e010779.
Pal S, Shekhar R, Kottewar S, Upadhyay S, Singh M, Pathak D, et al. COVID-19 Vaccine Hesitancy and Attitude toward Booster Doses among US Healthcare Workers. Vaccines. 2021;9:1358.
Janssen C, Maillard A, Bodelet C, Claudel A-L, Gaillat J, Delory T, et al. Hesitancy towards COVID-19 Vaccination among Healthcare Workers: A Multi-Centric Survey in France. Vaccines. 2021;9:547
Toth-Manikowski SM, Swirsky ES, Gandhi R, Piscitello G. COVID-19 vaccination hesitancy among health care workers, communication, and policy-making. Am J Infect Control. 2022;50:20-5.

**Reviewer**	**2**
**Name**	**Livia Asan**
**Affiliation**	**University Hospital Essen**
**Date**	**13-Jan-2025**
**COI**	**None**

My expertise lies with effects of communication and expectation also in the context of vaccinations, but I have no expertise with the methodology of mixed-methods analysis and, in particular, qualitative investigations. Therefore, I focused my assessment on quantitative methodology and all other aspects of this work including the study rationale, design, presentation of data, evaluation of results, discussion, and conclusions.

Summary
The work titled "Understanding barriers and enablers for vaccination against COVID-19 and influenza amongst healthcare workers in the SIREN Study" by Sparkes et al. presents data on vaccine coverage among health-care workers (HCW) in the UK which participated in the SIREN study. The SIREN study is a large prospective observational cohort study with an impressive sample size conducted at several study centers across the UK recruiting health

care workers (HCWs) with the primary goal to determine whether the presence of antibodies against SARS-CoV2 is associated with a reduction in the subsequent risk of re-infection. Purpose of this present investigation was to present data on vaccination coverage against COVID-19 (C-19) and influenza across the course of the C-19 pandemic in this study cohort of n= 6,048 participants, and to dissect vaccination coverage for different demographic subgroups as defined by age, ethnicity, occupation and gender. They compare their findings here with the National surveillance data for HCW in England, which exhibits less granular resolution of the demographics than the cohort studied here. Following the quantitative analysis of vaccine coverage, the authors designed and conducted a qualitative investigation using focus groups and semi-structured interviews. In the focus groups, researchers attempted to construct a group which represents the demographic characteristics from the whole dataset. Aim was to explore systemic and personal/individual enablers and barriers to receiving vaccinations. One focus was also to explore potential influences between the decision to receive the C-19 and/or influenza vaccine. After grouping conceptionally separate aspects using coding of transriptions and construction of themes, the authors present illustrative quotes from the interviews and focus groups for enablers and barriers. From this, the authors derive recommendations to address enablers and barriers in future vaccination rollouts.

Strengths:
Investigating vaccine coverage and potential barriers and enablers for vaccination are especially crucial in this population of HCW, as they are at increased infection risk and can contribute to spreading infection within vulnerable populations, and, due to this, are prioritized in vaccine rollouts and also function as a model role for the rest of the population for vaccine acceptance. Focusing on this group of people certainly makes sense. Furthermore, the analysis of subgroups can help decision-makers such as politicians and leaders in health-care to target groups that are at risk of facing more barriers, and promote measures that enable vaccination. The mixed-methods approach seems suitable and valuable since hypothetic reasons for choices regarding vaccination throughout the pandemic can be assessed first hand from within the study collective. The usage of adjusted odds ratios from multivariable logistic regression modeling is suitable and intelligable for meaningful descriptive comparisons between groups. The observation throughout the pandemic with three "vaccination seasons" allows one to inspect dynamics in vaccination coverage over time, and it is also of high value to explore potential direct and indirect influences between two very relevant vaccinations – against C-19 and against influenza – with the goal to understand and in the future address enablers that promote receiving both these important preventative treatments.
Limitations and drawbacks:
Since the SIREN study schedule included common (fortnightly) direct contacts with study personnel and the participants were asked about their vaccination status each time, which probably enhances attention towards the topic and increases incentive to do the socially

desirable choice and get the vaccination. The authors do adequately address this potential confound in their quantitative analysis already.

Especially to the seemingly small sample size of the qualitative analysis, specific barriers that minorities potentially face could not be adequately assessed, which the authors mention themselves in critical reflection.

Assessing motives in favor or against vaccination throughout a past time span of several years in retrospective in the qualitative analysis has drawbacks and biases in comparison to a prospective or accompanying evaluation.

The research question of this particular work has not been preregistered in detail, and accordingly, the authors refrain from formulating and addressing specific a priori hypotheses. However, within this hypothesis-generating study, the results from this qualitative analysis are instructive to develop targeted questionnaires designed to test hypotheses about motives, enablers and barriers during future vaccination rollouts.

Several suggested points that could still be addressed to even further improve the clarity and accuracy of the manuscript are listed below:

Comments:

I believe a closer inspection of this rich and valuable longitudinal dataset could even provide some more basis for the authors' proposed themes of enablers and barriers for vaccination from the qualitative analysis. In particular, I would like to suggest two (descriptive/exploratory) additional analyses, which I believe could strengthen the manuscript. If the authors have reasons not to perform the additional analyses, it would still be helpful to have the authors' feedback on these questions and requests.

1) With reference to the participants statement of being hesitant to receive another C-19 shot, "knowing that the COVID vaccine will give me a miserable 24 hours..": If data on side effects after vaccinations is available from the SIREN study, can the authors see if there is any indication that the experience of (mild) side effects after vaccination influenced later utilization of vaccinations (both C-19 and flu)? This could be interesting since negative treatment experiences and generalizations could be directly addressed, e.g. through communication that emphasizes coping strategies and uses positive side effect reframing (Crum, Heathcote et al. 2023).

2) Maybe it could be worth investigating the dataset for indicators concerning their assumption that offering both flu and COVID-19 vaccinations together could have motivated some HCWs to receive the C-19 booster. For example, the authors could add a pie chart or similar of all C-19 booster recipients (booster 1 and 2 separately), showing what percentage reported to have received that year's seasonal flu shot at the same study timepoint, or before their COVID-19-shot, or after it, or which percentage did not receive the C-19 booster at all. Same could be done viceversa (how many of the flu recipients of each season have received their C-19-booster at the same time, prior, after their flu shot, or not at all?). The result could be interesting and also show how large the overlap between C-19 and flu shot recipients is (which has not been addressed yet).

Other points:
1) One striking observation in the SIREN cohort is that vaccination coverage against influenza showed to increase from season to season during the pandemic, while the nationwide data clearly showed a decrease in vaccine coverage. The context that could have led to this discrepancy in vaccination behavior could be addressed more thoroughly. In particular, the authors could provide more details about the content of communication that they provided the SIREN study participants about each vaccine program in the beginning and throughout the study, and if their consumption was monitored as part of the study protocol or not. Was there a particular content or type of media that could have been especially effective at conveying a positive take towards the vaccination and that the authors would be willing to describe in more detail in order to inform future efforts to enable vaccination coverage?

2) Adding to this, the first four out of eight of the authors recommendations (Box 1) call for improving communication as a route to enhance enablers and reduce barriers. However, they give no information on how this communication should look like. This is important since we know from studies (Faasse, Porsius et al. 2017, Tan, Colagiuri et al. 2022, Asan, Kleine-Borgmann et al. 2024) that media coverage about vaccination side effects can also promote unwanted nocebo effects (= negative health outcomes through negative expectations) and contribute to vaccination hesitancy (Rief 2021). A link between nocebo effects and vaccination hesitancy has also been reported in HCWs (Mitsikostas, Aravantinou-Fatorou et al. 2021). Consequently, it is most probably not only important to communicate MORE, but also to communicate RIGHT. There is a growing body of literature about tested communication strategies on vaccines and other treatments (MacKrill, Morrison et al. 2021, Mao, Barnes et al. 2021, Clemens, Faasse et al. 2023) and recommendations (Bingel and Placebo Competence 2014, Rief 2021) intended to increase desirable effects and avoid nocebo effects, e.g. through positive framing, emphasizing coping strategies, using observational learning, informing about nocebo effects, adapting an empathetic and authentic communication style etc. Reflecting these considerations in their recommendations in the text and ideally relating these to their own communication during the SIREN study could refine and concretize their conclusions, make them more helpful and goal-directed, and embed their work into current knowledge on these aspects of communication in health care.

3) I'd like to ask the authors should specify their statement "In this study, the influenza vaccine was more widely acceptable compared with the COVID-19 vaccine due to the availability of more data" (bottom of page 20). Which availability (from whom? Study personnel, general media?) of which data (disease severity? Vaccine safety?) is being referred to here?

4) The authors seem to have used National surveillance data on HCW vaccination uptake in England only for comparison, whereas the SIREN cohort includes participants from sites all over the UK. It would be nice if the authors could explain their choice to focus on England shortly as an answer to this comment and briefly state if there is reason to assume that other UK countries have largely different vaccine coverage numbers than England.

5) Page 7: “Vaccine coverage (the proportion of the cohort vaccinated) was calculated for each of the four COVID-19 vaccines”. For clarity and precision, please specify which four COVID-19 vaccines are meant, as there are more than four COVID-19 vaccines used globally and which the readers will be more or less familiar with.

6) Table 2 says “lastminute.com” in one of the quotes. I assume that’s a typo.

7) There was no Figure 3 in the transmitted PDF (referred to at the bottom of page 10).

Lastly, I would like to congratulate the authors and for their effort to illustrate differences in vaccination coverage across these different demographic and occupational groups, and to identify enablers and barriers in this impressive dataset of this large undertaking in the SIREN study. The manuscript is of high relevance to the fields of preventive medicine, public health and medical psychology. The manuscript is generally recommended for publication but I suggest to address the mentioned points in an effort to support their statements and improve clarity. I thank the authors and the journal for the opportunity to review this manuscript.

References:
Asan, L., J. Kleine-Borgmann, B. Bozkurt, B. Frank, M. Kohrmann, C. Kleinschnitz and U. Bingel (2024). "Media coverage of COVID-19 vaccination-associated cerebral venous sinus thrombosis was followed by a surge in emergency presentations due to headache - observations from a university hospital in Germany." Front Psychiatry 15: 1378472.
Bingel, U. and T. Placebo Competence (2014). "Avoiding nocebo effects to optimize treatment outcome." JAMA 312(7): 693-694.
Clemens, K. S., K. Faasse, W. Tan, B. Colagiuri, L. Colloca, R. Webster, L. Vase, E. Jason and A. L. Geers (2023). "Social communication pathways to COVID-19 vaccine side-effect expectations and experience." J Psychosom Res 164: 111081.
Crum, A. J., L. C. Heathcote, Z. Morrison, R. Yielder, K. Leibowitz, H. Petousis-Harris, M. G. Thomas, C. G. Prober, J. S. Berek and K. J. Petrie (2023). "Changing Mindsets About Side Effects of the COVID-19 Vaccination: A Randomized Controlled Trial." Ann Behav Med 57(11): 901-909.
Faasse, K., J. T. Porsius, J. Faasse and L. R. Martin (2017). "Bad news: The influence of news coverage and Google searches on Gardasil adverse event reporting." Vaccine 35(49 Pt B):

6872-6878.
MacKrill, K., Z. Morrison and K. J. Petrie (2021). "Increasing and dampening the nocebo response following medicine-taking: A randomised controlled trial." J Psychosom Res 150: 110630.
Mao, A., K. Barnes, L. Sharpe, A. L. Geers, S. G. Helfer, K. Faasse and B. Colagiuri (2021). "Using Positive Attribute Framing to Attenuate Nocebo Side Effects: A Cybersickness Study." Ann Behav Med 55(8): 769-778.
Mitsikostas, D. D., K. Aravantinou-Fatorou, C. Deligianni, E. Kravvariti, E. Korompoki, M. Mylona, P. Vryttia, G. Papagiannopoulou, E. M. Delicha, A. Dellis, G. Tsivgoulis, M. A. Dimopoulos, M. Amanzio and P. P. Sfikakis (2021). "Nocebo-Prone Behavior Associated with SARS-CoV-2 Vaccine Hesitancy in Healthcare Workers." Vaccines (Basel) 9(10).
Rief, W. (2021). "Fear of Adverse Effects and COVID-19 Vaccine Hesitancy: Recommendations of the Treatment Expectation Expert Group." JAMA Health Forum 2(4): e210804.
Tan, W., B. Colagiuri and K. Barnes (2022). "Factors Moderating the Link between Personal Recounts of COVID-19 Vaccine Side Effects Viewed on Social Media and Viewer Postvaccination Experience." Vaccines (Basel) 10(10).

**Reviewer**	**3**
**Name**	**Joshuan Barboza**
**Affiliation**	**Universidad Cesar Vallejo**
**Date**	**13-Jan-2025**
**COI**	**None**

The study is interesting, however, I would suggest adding the objectives or the projection of the study to be consistent with the results and the conclusion

---

**Reviewer**	**4**
**Name**	**Nebiyu Dereje**
**Affiliation**	**Wachemo University, Addis Ababa University College of Health Sciences**
**Date**	**13-Jan-2025**
**COI**	**None**

Thank you for the opportunity to review a manuscript entitled “Understanding barriers and enablers for vaccination against COVID-19 and influenza amongst healthcare workers in the SIREN Study”. This manuscript highlights the vaccines coverage among HCWs and explores the barriers and enablers for the vaccine uptake. The following are my comments to improve the quality of the manuscript.

1. Page 2, Line 45 – 49: The statement “Participants were representative of the HCW workforce” should not be included here. The authors can reflect on this in the discussion.
2. Page 2, Line 45 – 49: …..with the majority being female (83%) and of white-ethnicity (88%). Nurses made up the largest occupational group (33%). These are the study findings and are best suited in the results section.
3. Page 3, Lines 16 -21: Please include 95% confidence intervals for the coverages (%).
4. Page 3: Results – No summary findings from the multivariable analysis have been stated.
5. Page 4: Summary box: As per the journal’s guidelines, this section is meant for the methodological strengths and limitations of the study. Please revise accordingly.
6. Page 5: Introduction: Lines 29 – 33: During the initial roll out of COVID-19 vaccines, uptake amongst HCWs was unprecedented with 91.2% of HCWs receiving at least one dose and 88.5% receiving two but with declining uptake for third and subsequent doses(9). Please add place and time to this statement.
7. The introduction needs to justify why this study was conducted. It should clearly indicate the gap in previous evidence (knowledge) that this study intended to fill. Please revise the introductions' last two paragraphs to justify the need for this study.
8. Objective a) compare trends in COVID-19 and influenza vaccine coverage throughout the pandemic in the SIREN cohort ----please add ‘among HCWs’
9. Please remove “using a sequential mixed methods approach” from the objective…this is repetitive.
10. Write the mixed methods function as [QUANI qual]
11. The authors need to provide more design information on the cohort study
12. The authors need to specify the type of study design used for the qualitative part of the mixed methods study
13. The sampling and sample size determination procedures are not clear. Please provide detailed information on the sampling procedures followed. How was recruitment for the quantitative and qualitative parts handled?
14. Please include data collection methods used by the type of study design employed. How was the quantitative data collected? How was the qualitative data collected? It is not clear as it is written in the manuscript.
15. Participants were offered an honorarium of a £50 voucher for participation in line with NIHR guidance ….how do you see this incentivization in light of existing ethical principles? Is it ethically recommended to provide incentives for study participants?
16. Were data collected recorded? Who were FGD participants? How was the discussion handled? How familiar were the facilitators? What was their role during the discussion? How long did the FGD and interview take? How were the data collection tools validated? Was there piloting of the tools?
17. How was the trustworthiness (rigor) of qualitative data assured?
18. The data analysis section speaks only about the qualitative part of the study. How was the quantitative part of the study analyzed? How were the descriptive and analytical components of this data handled? What was the outcome variable? How was it measured?

What were the explanatory variables? How were the explanatory variables measured? What effect measure was used? How was the multivariable analysis conducted?
19. Results: Please include 95% CI for the coverage estimates.
20. Some of the statements included in the results should be kept for the discussion section. For example, 'The trend of decreasing coverage of COVID-19 vaccinations in SIREN is consistent with national data, although coverage in SIREN has been consistently higher than observed nationally. National coverage of the 2nd COVID-19 booster in HCW decreased from 88% (primary course), to 68% (1st booster) to 42% (2nd booster), (7, 9, 23). In contrast to SIREN, national data on influenza vaccination coverage declined nationally with 49% vaccinated in 2022/23 compared to 61% in 2021/22 and 77% in 2020/21. National influenza vaccination coverage in HCWs for 2022/23 was substantially below the target of 75% for clinical staff set via the Commissioning for Quality and Innovation (CQUIN) framework (24), and the lowest recorded since the introduction of the CQUIN in 2023. (23) There is marked variability in coverage of both vaccines by NHS trust. The results section is for a concise and precise write-up of your findings and no reference citations are included. Please move this paragraph to the discussion section.
21. Please move this to the discussion 'National surveillance data is aggregated by 'doctors', 'nurses', 'other clinical' and 'support' staff groups. This contrasts with the more detailed occupational data available in SIREN, and likely obscures heterogeneity within groups. In particular, the 'other clinical' group includes therapists, HCAs and estates staff/porters who had variable coverage in the SIREN data.'
22. The interpretation of the adjusted odds ratio needs to be included in the multivariable analysis section.
23. Please thematize the sub-themes based on similarities and differences (Table 2). Some of the sub-themes can be merged.
24. Barriers and enablers related to the health systems, individual characteristics, governance and policy…were not optimally explored or described.
25. Discussion – please discuss the quantitative coverage findings, including their clinical and public health implications.
26. Limitations – The representativeness of the study participants has been indicated in this manuscript both as a strength (see introduction) and a limitation (see discussion). This is inconsistent. Please revise this.
27. This study has more potential limitations: online interviews and FGD, selection bias due to sampling errors, information bias (social-desirability bias), and researcher bias in qualitative studies…
28. ….design – focus groups and in-depth interviews – these are not study designs, they are data collection methods.
29. Figure 2 can better be presented in a multivariable Table to provide the actual OR values along with 95% confidence intervals.

VERSION 1 - AUTHOR RESPONSE

Reviewer: 1
Dr. Anastasia Saade, Université de Rennes, IRSET
Comments to the Author:
Thank you for the opportunity to review this manuscript. This topic is of interest and indeed few studies addressed the attitude of HCWs towards influenza and COVID booster vaccination in the COVID post-pandemic era.
The strength of this work is clearly the sample size with interesting data on vaccination coverage kinetics from 2020 to 2023.
We expect new data of vaccine hesitancy provided the very big number of participants. However, this paper does not provide with something original concerning the quantitative part of this study. The qualitative data, which are much appreciate nowadays, despite its small sample size provides very interesting data, highlighting the lack of information on vaccine risks and benefits.

Major comments :
The manuscript could probably be written in a different manner to make it more fluent.
Many thanks for the comment we have gone back through the manuscript to try to ensure any errors have been removed and that the sentences flow more clearly to make the manuscript more fluent.

You state that your study adds : identification of HCWs groups with high and low vaccine coverage. This question was already reported in a couple of papers in the literature showing that doctors much often receive influenza annual vaccination compared to nurses or nurse assistants.
Thank you, we agree that there have been other studies which show that doctors have higher vaccine coverage than nurses, however, we feel that the inclusion of other professionals, including therapists, pharmacists, porters, midwives and HCAs and comparing them does bring new insights and in much greater depth than previously documented.

Methods : Do you use a multivariable analysis ? or a multivariate analysis ? Please state the software used for your statistical analysis.
We use multivariable analysis and have included the software we used for the analysis.

Would cluster analysis be more stringent ?
Thank you for the proposed methodological change, however, we believe that a multivariable analysis is the appropriate methodology for the quantitative section and thematic analysis for the qualitative section. Whilst participants were recruited from multiple centres originally, at this stage of the study the numbers of participants per site is highly variable, participants may have moved workplace, and therefore a cluster analysis

based on site would not be appropriate.

Wasn't COVID vaccination compulsory for HCWs? if this was the case please state it in the introduction.
COVID vaccination was never made compulsory in the UK for HCWs, whilst it was discussed as a possibility, it was never introduced.

Your quantitative analysis does not provide data on barriers and facilitators but rather the qualitative data alone. Please temper these sentences in your manuscript.
Thank you for your comment, I have added that we have identified barriers through focus groups where we talk about barriers and facilitators to ensure clarity.

Qualitative data do not distinguish between HCWs categories ? why didn't you provide with this data ?
The HCWs were selected to be broadly representative of the different groups and we do provide demographic details in supplementary 2, however, to maintain anonymity of participants we did not distinguish between the HCW categories

Minor comments :
Introduction
Line 45 : add a comma after "infections and mortality"
Many thanks, this has been added

Methods
Page 6 Line 22 : "A mixed-methods study nested within the SIREN multicentre prospective cohort study of HCWs across the UK" please add a verb to the sentence
Thanks for the comment, I have added, "was used" to the end for clarity

Page 6 Line 48 :"COVID-19 and influenza vaccine data (date and type) was obtained" ==> were obtained ?
Thank you, I have amended this

Page 7 line 20 : "year, in line with dates HCWs can receive each vaccine)" : I do not understand that sentence.
HCWs in the UK are only able to receive influenza vaccines between September and March each year, so that is how we defined the different seasons, which we felt needed to be clarified as it is different to how the COVID-19 vaccines where rolled out, and so we used which vaccine booster they received.

Page 7 : I am wondering whether this paragraph would better fit in the study design section "Vaccine coverage (the proportion of the cohort vaccinated) was calculated for each of the

four
COVID-19 vaccines, and for each influenza season (defined as 1st September until 31st
March each
year, in line with dates HCWs can receive each vaccine) between September 2020 and March
2023
(3 seasons). For COVID-19 boosters, the 1st booster season was defined as 01/09/2021-
31/03/2022,
and the 2nd booster as 01/09/2022-31/03/2023."
We consider it fits best in the data analysis section as this is a calculated measure and this paragraph describes the methodology used.

References :
Please discuss

Mohr NM, Plumb ID, Santos León E, Harland KK, Krishnadasan A, Nandi U, et al. Factors associated with the decision to receive bivalent COVID-19 booster vaccination among health care personnel. Hum Vaccines Immunother. 2023;19:2284471.

SteelFisher GK, Findling MG, Caporello HL, McGowan E, Espino L, Sutton J. Divergent Attitudes Toward COVID-19 Vaccine vs Influenza Vaccine. JAMA Netw Open.2023;6:e2349881.

Kessy SJ, Wei T, Zhou Y, Zhang W-X, Alwy Al-Beity FM, Zhang S-S, et al. Vaccination willingness, vaccine hesitancy, and estimated coverage of SARS-CoV-2 vaccine among healthcare workers in Tanzania: A call for action. Immun Inflamm Dis. 2023;11:e1126.

Choi Y, Park S, Lee J, Kim Y, Kim BJ, Lin L, et al. Who gets COVID-19 booster vaccination? Trust in public health institutions and promotion strategies post-pandemic in the Republic of Korea. Vaccine. 2023;41:7560-72.

Durando P, Alicino C, Dini G, Barberis I, Bagnasco AM, Iudici R, et al. Determinants of adherence to seasonal influenza vaccination among healthcare workers from an Italian region: results from a cross-sectional study. BMJ Open. 2016;6:e010779.

Pal S, Shekhar R, Kottewar S, Upadhyay S, Singh M, Pathak D, et al. COVID-19 Vaccine Hesitancy and Attitude toward Booster Doses among US Healthcare Workers. Vaccines. 2021;9:1358.

Janssen C, Maillard A, Bodelet C, Claudel A-L, Gaillat J, Delory T, et al. Hesitancy towards COVID-19 Vaccination among Healthcare Workers: A Multi-Centric Survey in France. Vaccines. 2021;9:547

Toth-Manikowski SM, Swirsky ES, Gandhi R, Piscitello G. COVID-19 vaccination hesitancy among health care workers, communication, and policy-making. Am J Infect Control. 2022;50:20-5.

Thank you for including these suggestions. We have now included some of these papers in the discussion.

Reviewer: 2
Dr. Livia Asan, University Hospital Essen
Comments to the Author:
Comment to Authors
My expertise lies with effects of communication and expectation also in the context of vaccinations, but I have no expertise with the methodology of mixed-methods analysis and, in particular, qualitative investigations. Therefore, I focused my assessment on quantitative methodology and all other aspects of this work including the study rationale, design, presentation of data, evaluation of results, discussion, and conclusions.

Summary
The work titled "Understanding barriers and enablers for vaccination against COVID-19 and influenza amongst healthcare workers in the SIREN Study" by Sparkes et al. presents data on vaccine coverage among health-care workers (HCW) in the UK which participated in the SIREN study. The SIREN study is a large prospective observational cohort study with an impressive sample size conducted at several study centers across the UK recruiting health care workers (HCWs) with the primary goal to determine whether the presence of antibodies against SARS-CoV2 is associated with a reduction in the subsequent risk of re-infection. Purpose of this present investigation was to present data on vaccination coverage against COVID-19 (C-19) and influenza across the course of the C-19 pandemic in this study cohort of n= 6,048 participants, and to dissect vaccination coverage for different demographic subgroups as defined by age, ethnicity, occupation and gender. They compare their findings here with the National surveillance data for HCW in England, which exhibits less granular resolution of the demographics than the cohort studied here. Following the quantitative analysis of vaccine coverage, the authors designed and conducted a qualitative investigation using focus groups and semi-structured interviews. In the focus groups, researchers attempted to construct a group which represents the demographic characteristics from the whole dataset. Aim was to explore systemic and personal/individual enablers and barriers to receiving vaccinations. One focus was also to explore potential influences between the decision to receive the C-19 and/or influenza vaccine. After grouping conceptionally separate aspects using coding of transriptions and construction of themes, the authors present illustrative quotes from the interviews and focus groups for enablers and barriers. From this, the authors derive recommendations to address enablers and barriers in future vaccination

rollouts.

Strengths:
Investigating vaccine coverage and potential barriers and enablers for vaccination are especially crucial in this population of HCW, as they are at increased infection risk and can contribute to spreading infection within vulnerable populations, and, due to this, are prioritized in vaccine rollouts and also function as a model role for the rest of the population for vaccine acceptance. Focusing on this group of people certainly makes sense. Furthermore, the analysis of subgroups can help decision-makers such as politicians and leaders in health-care to target groups that are at risk of facing more barriers, and promote measures that enable vaccination. The mixed-methods approach seems suitable and valuable since hypothetic reasons for choices regarding vaccination throughout the pandemic can be assessed first hand from within the study collective. The usage of adjusted odds ratios from multivariable logistic regression modeling is suitable and intelligable for meaningful descriptive comparisons between groups. The observation throughout the pandemic with three “vaccination seasons” allows one to inspect dynamics in vaccination coverage over time, and it is also of high value to explore potential direct and indirect influences between two very relevant vaccinations – against C-19 and against influenza – with the goal to understand and in the future address enablers that promote receiving both these important preventative treatments.

Limitations and drawbacks:
Since the SIREN study schedule included common (fortnightly) direct contacts with study personnel and the participants were asked about their vaccination status each time, which probably enhances attention towards the topic and increases incentive to do the socially desirable choice and get the vaccination. The authors do adequately address this potential confound in their quantitative analysis already.

Especially to the seemingly small sample size of the qualitative analysis, specific barriers that minorities potentially face could not be adequately assessed, which the authors mention themselves in critical reflection.

Assessing motives in favor or against vaccination throughout a past time span of several years in retrospective in the qualitative analysis has drawbacks and biases in comparison to a prospective or accompanying evaluation.
The research question of this particular work has not been preregistered in detail, and accordingly, the authors refrain from formulating and addressing specific a priori hypotheses. However, within this hypothesis-generating study, the results from this qualitative analysis are instructive to develop targeted questionnaires designed to test hypotheses about motives, enablers and barriers during future vaccination rollouts.
Several suggested points that could still be addressed to even further improve the clarity and

accuracy of the manuscript are listed below:

Comments:
I believe a closer inspection of this rich and valuable longitudinal dataset could even provide some more basis for the authors' proposed themes of enablers and barriers for vaccination from the qualitative analysis. In particular, I would like to suggest two (descriptive/exploratory) additional analyses, which I believe could strengthen the manuscript. If the authors have reasons not to perform the additional analyses, it would still be helpful to have the authors' feedback on these questions and requests.

1) With reference to the participants statement of being hesitant to receive another C-19 shot, "knowing that the COVID vaccine will give me a miserable 24 hours..": If data on side effects after vaccinations is available from the SIREN study, can the authors see if there is any indication that the experience of (mild) side effects after vaccination influenced later utilization of vaccinations (both C-19 and flu)? This could be interesting since negative treatment experiences and generalizations could be directly addressed, e.g. through communication that emphasizes coping strategies and uses positive side effect reframing (Crum, Heathcote et al. 2023).
We completely agree that this is very important information and would be very useful. We did not collect data on side effects following vaccination in the original SIREN study questionnaire as it was not one of it's initial primary aims. However, having seen that this data would be useful from this research we have sent out an additional questionnaire to participants to explore side effects, amongst other issues related to HCW attitudes towards vaccination here, this will be published as a standalone article in due course.

2) Maybe it could be worth investigating the dataset for indicators concerning their assumption that offering both flu and COVID-19 vaccinations together could have motivated some HCWs to receive the C-19 booster. For example, the authors could add a pie chart or similar of all C-19 booster recipients (booster 1 and 2 separately), showing what percentage reported to have received that year's seasonal flu shot at the same study timepoint, or before their COVID-19-shot, or after it, or which percentage did not receive the C-19 booster at all. Same could be done viceversa (how many of the flu recipients of each season have received their C-19-booster at the same time, prior, after their flu shot, or not at all?). The result could be interesting and also show how large the overlap between C-19 and flu shot recipients is (which has not been addressed yet).

We agree this would have been interesting, however, the comment is from the qualitative research rather than the quantitative and the qualitative element of the study was conducted after the quantitative so we would not be able to reliably get this data. However, as above, we completely agree that this is interesting and valuable data, and so have included this approach in the questionnaire which we have designed for the next article.

Other points:
1) One striking observation in the SIREN cohort is that vaccination coverage against influenza showed to increase from season to season during the pandemic, while the nationwide data clearly showed a decrease in vaccine coverage. The context that could have led to this discrepancy in vaccination behavior could be addressed more thoroughly. In particular, the authors could provide more details about the content of communication that they provided the SIREN study participants about each vaccine program in the beginning and throughout the study, and if their consumption was monitored as part of the study protocol or not. Was there a particular content or type of media that could have been especially effective at conveying a positive take towards the vaccination and that the authors would be willing to describe in more detail in order to inform future efforts to enable vaccination coverage?
Thank you for this comment, we have tried to clarify what we meant here in the text more clearly. During the period of the study we were expanding the scope of the SIREN study to include investigation of influenza and RSV in addition to COVID-19, to keep our participants informed we explained clearly the rationale for this – including why influenza and RSV are important viruses. This may have encouraged participants to get the influenza vaccine and so explain the increased uptake rather than decrease, which certainly we are seeing more widely.

2) Adding to this, the first four out of eight of the authors recommendations (Box 1) call for improving communication as a route to enhance enablers and reduce barriers. However, they give no information on how this communication should look like. This is important since we know from studies (Faasse, Porsius et al. 2017, Tan, Colagiuri et al. 2022, Asan, Kleine-Borgmann et al. 2024) that media coverage about vaccination side effects can also promote unwanted nocebo effects (= negative health outcomes through negative expectations) and contribute to vaccination hesitancy (Rief 2021). A link between nocebo effects and vaccination hesitancy has also been reported in HCWs (Mitsikostas, Aravantinou-Fatorou et al. 2021). Consequently, it is most probably not only important to communicate MORE, but also to communicate RIGHT. There is a growing body of literature about tested communication strategies on vaccines and other treatments (MacKrill, Morrison et al. 2021, Mao, Barnes et al. 2021, Clemens, Faasse et al. 2023) and recommendations (Bingel and Placebo Competence 2014, Rief 2021) intended to increase desirable effects and avoid nocebo effects, e.g. through positive framing, emphasizing coping strategies, using observational learning, informing about nocebo effects, adapting an empathetic and authentic communication style etc. Reflecting these considerations in their recommendations in the text and ideally relating these to their own communication during the SIREN study could refine and concretize their conclusions, make them more helpful and goal-directed, and embed their work into current knowledge on these aspects of communication in health care.
We could not agree more with this very important point. I have added some lines into the

discussion highlighting the influence of negative media press on side effects and have added "positive" messaging into our recommendations, as whilst we do believe that it is important to be open and honest with HCWs with the statistics on side effects to gain trust, framing it in a positive manner is beneficial.

3) I'd like to ask the authors should specify their statement "In this study, the influenza vaccine was more widely acceptable compared with the COVID-19 vaccine due to the availability of more data" (bottom of page 20). Which availability (from whom? Study personnel, general media?) of which data (disease severity? Vaccine safety?) is being referred to here?

Thanks, have tried to clarify, we meant that they were more positive as they are more familiar with it, particularly the long term side effects as there is more evidence that there are not long term side effects.

4) The authors seem to have used National surveillance data on HCW vaccination uptake in England only for comparison, whereas the SIREN cohort includes participants from sites all over the UK. It would be nice if the authors could explain their choice to focus on England shortly as an answer to this comment and briefly state if there is reason to assume that other UK countries have largely different vaccine coverage numbers than England.

Thanks, we would have liked to include all data, but only England publishes this data, so we could not compare with the Devolved Nations.

5) Page 7: "Vaccine coverage (the proportion of the cohort vaccinated) was calculated for each of the four COVID-19 vaccines". For clarity and precision, please specify which four COVID-19 vaccines are meant, as there are more than four COVID-19 vaccines used globally and which the readers will be more or less familiar with.

Apologies for the confusion, this was meant to mean the vaccine dose, rather than the specific vaccine type, as in initial course, followed by booster 1, booster 2 etc. I have changed this to "vaccine doses" for clarity.

6) Table 2 says "lastminute.com" in one of the quotes. I assume that's a typo.
Thanks, we are quoting verbatim, that is what the participant said, so it isn't a typo!

7) There was no Figure 3 in the transmitted PDF (referred to at the bottom of page 10).
This was a typo, should have said Figure 2

Lastly, I would like to congratulate the authors and for their effort to illustrate differences in vaccination coverage across these different demographic and occupational groups, and to identify enablers and barriers in this impressive dataset of this large undertaking in the SIREN study. The manuscript is of high relevance to the fields of preventive medicine, public health and medical psychology. The manuscript is generally recommended for publication but I suggest to address the mentioned points in an effort to support their statements and improve clarity. I thank the authors and the journal for the opportunity to review this manuscript.

Thank you very much for your kind words and contribution to the manuscript, we greatly appreciate it.

References:

Asan, L., J. Kleine-Borgmann, B. Bozkurt, B. Frank, M. Kohrmann, C. Kleinschnitz and U. Bingel (2024). "Media coverage of COVID-19 vaccination-associated cerebral venous sinus thrombosis was followed by a surge in emergency presentations due to headache - observations from a university hospital in Germany." Front Psychiatry 15: 1378472.
Bingel, U. and T. Placebo Competence (2014). "Avoiding nocebo effects to optimize treatment outcome." JAMA 312(7): 693-694.
Clemens, K. S., K. Faasse, W. Tan, B. Colagiuri, L. Colloca, R. Webster, L. Vase, E. Jason and A. L. Geers (2023). "Social communication pathways to COVID-19 vaccine side-effect expectations and experience." J Psychosom Res 164: 111081.
Crum, A. J., L. C. Heathcote, Z. Morrison, R. Yielder, K. Leibowitz, H. Petousis-Harris, M. G. Thomas, C. G. Prober, J. S. Berek and K. J. Petrie (2023). "Changing Mindsets About Side Effects of the COVID-19 Vaccination: A Randomized Controlled Trial." Ann Behav Med 57(11): 901-909.
Faasse, K., J. T. Porsius, J. Faasse and L. R. Martin (2017). "Bad news: The influence of news coverage and Google searches on Gardasil adverse event reporting." Vaccine 35(49 Pt B): 6872-6878.
MacKrill, K., Z. Morrison and K. J. Petrie (2021). "Increasing and dampening the nocebo response following medicine-taking: A randomised controlled trial." J Psychosom Res 150: 110630.
Mao, A., K. Barnes, L. Sharpe, A. L. Geers, S. G. Helfer, K. Faasse and B. Colagiuri (2021). "Using Positive Attribute Framing to Attenuate Nocebo Side Effects: A Cybersickness Study." Ann Behav Med 55(8): 769-778.
Mitsikostas, D. D., K. Aravantinou-Fatorou, C. Deligianni, E. Kravvariti, E. Korompoki, M. Mylona, P. Vryttia, G. Papagiannopoulou, E. M. Delicha, A. Dellis, G. Tsivgoulis, M. A. Dimopoulos, M. Amanzio and P. P. Sfikakis (2021). "Nocebo-Prone Behavior Associated with SARS-CoV-2 Vaccine Hesitancy in Healthcare Workers." Vaccines (Basel) 9(10).
Rief, W. (2021). "Fear of Adverse Effects and COVID-19 Vaccine Hesitancy: Recommendations of the Treatment Expectation Expert Group." JAMA Health Forum 2(4): e210804.

Tan, W., B. Colagiuri and K. Barnes (2022). "Factors Moderating the Link between Personal Recounts of COVID-19 Vaccine Side Effects Viewed on Social Media and Viewer Postvaccination Experience." Vaccines (Basel) 10(10).

Many thanks for these references, we have included some in the revised manuscript

Reviewer: 3
Dr. Joshuan Barboza, Universidad Cesar Vallejo
Comments to the Author:
The study is interesting, however, I would suggest adding the objectives or the projection of the study to be consistent with the results and the conclusion

Many thanks for the comment, I have adjusted the objectives (aims) to highlight the sequential nature of the study first investigating the quantitative vaccine coverage and moving onto the qualitative, barriers and facilitators which reflects the methods, results and then discussion points. The conclusion also now highlights first the inequality and then that we found barriers and made some recommendations.

Reviewer: 4
Dr. Nebiyu Dereje, Wachemo University, Addis Ababa University College of Health Sciences
Please also see attached file.

Comments to the Author:
Thank you for the opportunity to review a manuscript entitled “Understanding barriers and enablers for vaccination against COVID-19 and influenza amongst healthcare workers in the SIREN Study”. This manuscript highlights the vaccines coverage among HCWs and explores the barriers and enablers for the vaccine uptake. The following are my comments to improve the quality of the manuscript.

1. Page 2, Line 45 – 49: The statement “Participants were representative of the HCW workforce” should not be included here. The authors can reflect on this in the discussion.
Thank you, in the abstract it asks for a participants section for this journal so we included this information here – I am happy to move this if the editor would like us to?

2. Page 2, Line 45 – 49: …..with the majority being female (83%) and of white-ethnicity (88%). Nurses made up the largest occupational group (33%). These are the study findings and are best suited in the results section.
Thank you, in the abstract it asks for a participants section for this journal so we included this information here – I am happy to move this if the editor would like us to?

3. Page 3, Lines 16 -21: Please include 95% confidence intervals for the coverages (%).

Thank you for the comment, but these are observed figures rather than estimates, so there are no confidence intervals – they are descriptive statistics.

4. Page 3: Results – No summary findings from the multivariable analysis have been stated. Thank you, we included this summary "Two distinct groups emerged (Figure 2). Group one had high levels of vaccine coverage for both influenza and COVID-19, which included therapists, pharmacists and doctors and group two had low vaccine coverage for both influenza and COVID-19, which included healthcare assistants, estates staff/porters and participants in minority ethnic groups. There were no other groups where vaccine coverage was significantly higher for one vaccine than the other (Figure 2; Table S1). Overall influenza vaccine coverage was higher than COVID-19 in winter 2022/23, with no individual group accounting for this phenomenon."

5. Page 4: Summary box: As per the journal's guidelines, this section is meant for the methodological strengths and limitations of the study. Please revise accordingly.
We have amended this thank you.

6. Page 5: Introduction: Lines 29 – 33: During the initial roll out of COVID-19 vaccines, uptake amongst HCWs was unprecedented with 91.2% of HCWs receiving at least one dose and 88.5% receiving two but with declining uptake for third and subsequent doses(9). Please add place and time to this statement.
I have added in that it was UK and also that this ws by Feb 2022 as per the reference.

7. The introduction needs to justify why this study was conducted. It should clearly indicate the gap in previous evidence (knowledge) that this study intended to fill. Please revise the introductions' last two paragraphs to justify the need for this study.
Thank you I have tried to add in additional text to explain that the SIREN study is a unique cohort of HCWs and that this study allows us to see trends in a representative cohort and then explore these, which is a unique design at this scale.

8. Objective a) compare trends in COVID-19 and influenza vaccine coverage throughout the pandemic in the SIREN cohort ----please add 'among HCWs'
The SIREN cohort is all HCWs as explained in the paragraph before so we think that this is unnecessary repetition and we use "explore HCW perceptions" later in the sentence so think the repetition would sound repetitive.

9. Please remove "using a sequential mixed methods approach" from the objective…this is repetitive.
This is a recognised methodology and so we are keen to include this as it explains in the introduction how we are going to meet our aims and feel that this is useful for the reader –

however, if the editor feels that we should remove “using a mixed methods approach” then we are happy for it to be removed..

10. Write the mixed methods function as [QUANI qual]
I am sorry I don’t quite understand this comment

11. The authors need to provide more design information on the cohort study
We have introduced the cohort and provided references where the full methodology of the SIREN cohort is presented in view of the word count. We also present the demographic details within the body of the text

12. The authors need to specify the type of study design used for the qualitative part of the mixed methods study
Thank you we have amended the study design to be clearer highlighting that the qualitative part of the study was with focus groups with thematic analysis

13. The sampling and sample size determination procedures are not clear. Please provide detailed information on the sampling procedures followed. How was recruitment for the quantitative and qualitative parts handled?
Thanks for the question. We did not perform sampling size calculations for the quantitative analysis all participants were included which we have outlined in the participants section. I have expanded on the recruitment and selection of the qualitative participants to make it clearer, everybody was invited, screened to ensure representation from different groups, regions, ethnicity and ages.

14. Please include data collection methods used by the type of study design employed. How was the quantitative data collected? How was the qualitative data collected? It is not clear as it is written in the manuscript.
Thank you, we explained the data collection for quantitative data here:
“COVID-19 and influenza vaccine data (date and type) were obtained directly from participants via fortnightly follow-up surveys and through consented linkage on personal identifiers including name, date of birth and NHS number to the National Immunisation Management System (or equivalent system for the devolved administrations). Self-reported demographic data, including age, ethnicity, job role, were obtained from SIREN enrolment questionnaires sent between June 2020 and April 2021 National surveillance data on HCW vaccination uptake in England was obtained from the annual UKHSA official statistics(7).”
Qualitative data were collected by focus groups, I have added an additional sentence to clarify this

15. Participants were offered an honorarium of a £50 voucher for participation in line with NIHR guidance ….how do you see this incentivization in light of existing ethical principles? Is

it ethically recommended to provide incentives for study participants?
We followed the NIHR guidelines on using a honorarium which is standard practice for this type of research in the UK

16. Were data collected recorded? Who were FGD participants? How was the discussion handled? How familiar were the facilitators? What was their role during the discussion? How long did the FGD and interview take? How were the data collection tools validated? Was there piloting of the tools?
Thank you, we do mention that the data is recorded – they were recorded and transcribed verbatim and then the individual transcrips were allocated codes and then thematic analysis was conducted using Constant Comparison Analysis and provide the reference for this validated approach. We have added that the average length of the interviews was 90 minutes in the results section. I have added a sentence to explain that the facilitators had experience in facilitating focus groups. We used topic guides to aid facilitation as described. Finally, we did not do any piloting of the topic guides tools in advance.
Topic guide, average length 90 mins, facilitators independent of the study team but experienced

17. How was the trustworthiness (rigor) of qualitative data assured?
We assured this by using 2 researchers who were independent of the SIREN study team who, independently coded the transcripts and then met to determine discrepancies. Both researchers have been trained in facilitating and evaluating focus groups.

18. The data analysis section speaks only about the qualitative part of the study. How was the quantitative part of the study analyzed? How were the descriptive and analytical components of this data handled? What was the outcome variable? How was it measured? What were the explanatory variables? How were the explanatory variables measured? What effect measure was used? How was the multivariable analysis conducted?
There is a data analysis section for both the qualitative and quantitative sections, as they are sequential we have separated the 2 stages and have sections for each the participants, consent, data collection and data analysis.

19. Results: Please include 95% CI for the coverage estimates.
Thank you for the query but these are not estimates they are descriptive statistics.

20. Some of the statements included in the results should be kept for the discussion section. For example, 'The trend of decreasing coverage of COVID-19 vaccinations in SIREN is consistent with national data, although coverage in SIREN has been consistently higher than observed nationally. National coverage of the 2nd COVID-19 booster in HCW decreased from 88% (primary course), to 68% (1st booster) to 42% (2nd booster), (7, 9, 23). In contrast to SIREN, national data on influenza vaccination coverage declined nationally with 49%

vaccinated in 2022/23 compared to 61% in 2021/22 and 77% in 2020/21. National influenza vaccination coverage in HCWs for 2022/23 was substantially below the target of 75% for clinical staff set via the Commissioning for Quality and Innovation (CQUIN) framework (24), and the lowest recorded since the introduction of the CQUIN in 2023. (23) There is marked variability in coverage of both vaccines by NHS trust.
The results section is for a concise and precise write-up of your findings and no reference citations are included. Please move this paragraph to the discussion section.
Thank you, we have moved this to the discussion

21. Please move this to the discussion 'National surveillance data is aggregated by 'doctors', 'nurses', 'other clinical' and 'support' staff groups. This contrasts with the more detailed occupational data available in SIREN, and likely obscures heterogeneity within groups. In particular, the 'other clinical' group includes therapists, HCAs and estates staff/porters who had variable coverage in the SIREN data.'
I have moved this into the discussion, thanks.

22. The interpretation of the adjusted odds ratio needs to be included in the multivariable analysis section.
In view of brevity we explained the trends rather than listing the aORs as we feel they are clearly represented in the figure. Editor – we can add the numbers if you feel this is essential?

23. Please thematize the sub-themes based on similarities and differences (Table 2). Some of the sub-themes can be merged.
We thought quite carefully about the different sub-themes and would be keen to include these. I am unclear which should be combined, and it is difficult to combine them all into similarities and difference without losing some of the subtlety of the themes.

24. Barriers and enablers related to the health systems, individual characteristics, governance and policy…were not optimally explored or described.
Thank you, whilst we completely agree these are all very important and interesting barriers and enablers, they are beyond the scope of this study, and were not explored.

25. Discussion – please discuss the quantitative coverage findings, including their clinical and public health implications.
Thank you, we have amended the discussion to reflect this.

26. Limitations – The representativeness of the study participants has been indicated in this manuscript both as a strength (see introduction) and a limitation (see discussion). This is inconsistent. Please revise this.
Thank you, I have removed the representativeness in the introduction and kept – includes

“doctors, nurses, HCAs, therapists, midwives, pharmacists etc.

27. This study has more potential limitations: online interviews and FGD, selection bias due to sampling errors, information bias (social-desirability bias), and researcher bias in qualitative studies…
Thank you, I have added bits about selection and information bias. We feel that online interviews are acceptable as they became the standard way of conducting focus groups during the COVID-19 pandemic and it allowed participants from across the country to participate.

28. ….design – focus groups and in-depth interviews – these are not study designs, they are data collection methods.
Thank you, we have adjusted the study design section for clarity

29. Figure 2 can better be presented in a multivariable Table to provide the actual OR values along with 95% confidence intervals.

Thank you, we contest this as we believe that the figure is clearer for the reader and easier to appreciate visually than a table of numbers would be.

VERSION 2 - REVIEW

**Reviewer**	**1**
**Name**	**Anastasia Saade**
**Affiliation**	**Université de Rennes, IRSET**
**Date**	**13-Jan-2025**
**COI**	

Thank you for the opportunity to review your work. The combination of quantitative and qualitative data is enriching and adds depth to the analysis. While your findings largely reinforce what is already known about barriers and enablers to vaccination among healthcare workers, the large sample size strengthens the validity of your results and supports their generalizability to the broader UK healthcare workforce.

I would like to offer a few comments and suggestions that may help strengthen your manuscript.

Methods:
Why was R used only for graphical presentation and not for statistical analysis?

-The organization of the Methods section is somewhat confusing. The quantitative and qualitative components are presented separately, which makes it harder to follow. It would be clearer for the reader if the section were structured with unified subsections integrating both aspects.

- You mention that 6,048 participants were included—but out of how many eligible individuals?

- How many healthcare workers (HCWs) are included in the overall SIREN cohort?

- Do you have confidence intervals for the vaccination coverage rates?

- Have you considered presenting the data as prevalence and comparing prevalence rates across time periods?

- Comparisons across time periods may be difficult due to evolving contextual factors such as the emergence of new variants, changes in transmission and virulence, and shifts in attitudes toward vaccination. Did your multivariable analysis account for these temporal effects?

- Could you clarify how the themes were identified during the qualitative analysis?

- Were the interviews conducted using a standardized or semi-structured questionnaire?

- If so, is the questionnaire provided (e.g., in an appendix or supplementary material)?

Results:

- Is the SIREN cohort representative of the overall healthcare worker population in the UK?

- Could you provide absolute numbers in addition to percentages in the Results section?

- Were there any missing data, or did all 6,048 participants provide complete information?

- When referring to the "rate of uptake," do you mean rapid or slow? It would be helpful to define what constitutes a "rapid" versus "slow" uptake in your context.

- Would a cluster analysis be more appropriate to identify HCW profiles associated with vaccination behavior?

- The sentence "The primary course of COVID-19 vaccines were perceived as providing

protection…” should be revised to: *“The primary course of COVID-19 vaccines was perceived as providing protection…”*

- The Results section is quite long and could benefit from being shortened for clarity and readability.

Discussion :
I respectfully disagree with the recommendation to reduce the number of vaccination appointments by routinely co-administering COVID-19 and influenza vaccines. While the intent is to streamline the process, it is important to consider that these vaccines serve different purposes and often target different populations. Additionally, healthcare workers' attitudes toward each vaccine may differ. Offering only the combined vaccination could unintentionally discourage uptake of the influenza vaccine, and it is not certain that it would significantly improve COVID-19 vaccination coverage.

Given the small sample size of 24 healthcare workers, it may be challenging to develop robust guidelines based solely on these findings. A validation method, such as the Delphi technique, could be considered to strengthen and support the recommendations.

Was access to vaccination—such as location and scheduling—considered or assessed in the study?

Please add : Gaudouen, H., Tattevin, P., Thibault, V., Ménard, G., Paris, C., & Saade, A. (2024). Determinants of influenza and COVID vaccine uptake in healthcare workers: A cross-sectional survey during the post-pandemic era in a network of academic hospitals in France. Vaccine, 42(26), 126272. https://doi.org/10.1016/j.vaccine.2024.126272

**Reviewer**	**2**
**Name**	**Livia Asan**
**Affiliation**	**University Hospital Essen**
**Date**	**13-Jan-2025**
**COI**	

-

VERSION 2 - AUTHOR RESPONSE

Reviewer: 1
Dr. Anastasia Saade, Université de Rennes, IRSET
Comments to the Author:

Dear Authors,

Thank you for the opportunity to review your work. The combination of quantitative and qualitative data is enriching and adds depth to the analysis. While your findings largely reinforce what is already known about barriers and enablers to vaccination among healthcare workers, the large sample size strengthens the validity of your results and supports their generalizability to the broader UK healthcare workforce.

I would like to offer a few comments and suggestions that may help strengthen your manuscript.

Methods:
Why was R used only for graphical presentation and not for statistical analysis?

R was used rather than stata for graphical presentation as we felt the images produced look better, and stata was used for the statistical analysis as it is a programme that analysts were more familiar with. Both stata and R are standard programmes for both tools.

-The organization of the Methods section is somewhat confusing. The quantitative and qualitative components are presented separately, which makes it harder to follow. It would be clearer for the reader if the section were structured with unified subsections integrating both aspects.

Thank you for your comment, we respectfully disagree, the analysis was done sequentially and so we feel separating the methods in this way is easier to understand.

- You mention that 6,048 participants were included—but out of how many eligible individuals?

All 6,048 eligible participants were included I have further clarified this in the results

- How many healthcare workers (HCWs) are included in the overall SIREN cohort?

I have included the original cohort size in the introduction and clarified the number being 6,048 as above at the time of this study

- Do you have confidence intervals for the vaccination coverage rates?

These are observed figures and so there should not be confidence intervals, as explained in previous comments to reviewers

- Have you considered presenting the data as prevalence and comparing prevalence rates across time periods?

We considered this, but will be unable to make this change for this publication, we feel that how we have presented it is clear for readers.

- Comparisons across time periods may be difficult due to evolving contextual factors such as the emergence of new variants, changes in transmission and virulence, and shifts in attitudes toward vaccination. Did your multivariable analysis account for these temporal effects?

We explained what we take into account in the multivariable analysis, it would not be possible to account for changes in transmission and virulence. There are also a limited number of variants within a season and so we feel that what we have presented is acceptable

- Could you clarify how the themes were identified during the qualitative analysis?

This is included in the methods: "Focus groups and interviews were recorded and transcribed verbatim. Individual transcripts were allocated codes so they could not be identified. Thematic analysis was conducted on the transcripts using Constant Comparison Analysis"

- Were the interviews conducted using a standardized or semi-structured questionnaire?

Thank you, we used a topic guide which we will include this in the supplementary material

- If so, is the questionnaire provided (e.g., in an appendix or supplementary material)?

See above

Results:

- Is the SIREN cohort representative of the overall healthcare worker population in the UK?

Yes and we do discuss this in the discussion

- Could you provide absolute numbers in addition to percentages in the Results section?

The table already includes both the absolute number and the percentage

- Were there any missing data, or did all 6,048 participants provide complete information?

There was no missing data

- When referring to the "rate of uptake," do you mean rapid or slow? It would be helpful to define what constitutes a "rapid" versus "slow" uptake in your context.

There is no clear definition for this so I have amended it to say that uptake was faster, to make this clearer

- Would a cluster analysis be more appropriate to identify HCW profiles associated with vaccination behavior?

Thank you, as explained in the last comments to reviewers, we do not believe so.

- The sentence "The primary course of COVID-19 vaccines were perceived as providing protection..." should be revised to: *"The primary course of COVID-19 vaccines was perceived as providing protection..."*

Thank you we have changed this

- The Results section is quite long and could benefit from being shortened for clarity and readability.

Thank you, as there has been no comment as to what should be removed, and as such we shall keep it as it is and it was lengthened due to other reviewer comments previously

Discussion :
I respectfully disagree with the recommendation to reduce the number of vaccination appointments by routinely co-administering COVID-19 and influenza vaccines. While the intent is to streamline the process, it is important to consider that these vaccines serve different purposes and often target different populations. Additionally, healthcare workers' attitudes toward each vaccine may differ. Offering only the combined vaccination could unintentionally discourage uptake of the influenza vaccine, and it is not certain that it would significantly improve COVID-19 vaccination coverage.

Thank you for your comment, whilst you may disagree we are offering conclusions on our research and what our participants said, and so we would like to include this.

Given the small sample size of 24 healthcare workers, it may be challenging to develop robust guidelines based solely on these findings. A validation method, such as the Delphi technique, could be considered to strengthen and support the recommendations.

Thank you, this would be beyond the scope of this paper and we mention this as a limitation

Was access to vaccination—such as location and scheduling—considered or assessed in the study?

It was not possible to assess it during this study, however, we have in subsequent studies expanding on this work which have been published

Please add : Gaudouen, H., Tattevin, P., Thibault, V., Ménard, G., Paris, C., & Saade, A. (2024). Determinants of influenza and COVID vaccine uptake in healthcare workers: A cross-sectional survey during the post-pandemic era in a network of academic hospitals in France. Vaccine, 42(26), 126272. https://doi.org/10.1016/j.vaccine.2024.126272

I respectfully decline to add the reviewers work

Reviewer: 2
Dr. Livia Asan, University Hospital Essen
Comments to the Author:
-
